# Involvement of ACACA (acetyl-CoA carboxylase α) in the lung pre-metastatic niche formation in breast cancer by senescence phenotypic conversion in fibroblasts

**DOI:** 10.1007/s13402-022-00767-5

**Published:** 2023-01-06

**Authors:** Yung-Chi Huang, Ming-Feng Hou, Ying-Ming Tsai, Yi-Chung Pan, Pei-Hsun Tsai, Yi-Shiuan Lin, Chao-Yuan Chang, Eing-Mei Tsai, Ya-Ling Hsu

**Affiliations:** 1 Graduate Institute of Medicine, College of Medicine, Kaohsiung Medical University, No. 100, Shih-Chuan 1st Road, Kaohsiung, 807 Taiwan; 2 Department of Biomedical Science and Environment Biology, Kaohsiung Medical University, Kaohsiung, 807 Taiwan; 3 Division of Pulmonary and Critical Care Medicine, Kaohsiung Medical University Hospital, Kaohsiung, 807 Taiwan; 4 School of Medicine, College of Medicine, Kaohsiung Medical University, Kaohsiung, 807 Taiwan; 5 Drug Development and Value Creation Research Center, Kaohsiung Medical University, Kaohsiung, 807 Taiwan; 6 Department of Anatomy, Kaohsiung Medical University, Kaohsiung, 807 Taiwan; 7 Department of Biological Science and Technology, National Pingtung University of Science and Technology, Pingtung, 912 Taiwan

**Keywords:** Acetyl-CoA carboxylase α, Breast cancer, Metabolism reprogramming, Pre-metastatic niche, Senescence

## Abstract

**Background:**

Reprogramming of metabolism is strongly associated with the development of cancer. However, the role of metabolic reprogramming in the remodeling of pre-metastatic niche (PMN), a key step in metastasis, is still unknown. We aimed to investigate the metabolic alternation during lung PMN formation in breast cancer.

**Methods:**

We assessed the transcriptomes and lipidomics of lung of MMTV-PyVT mice by microarray and liquid chromatography-tandem mass mass spectrometry before lung metastasis. The validation of gene or protein expressions was performed by quantitative real-time polymerase chain reaction or immunoblot and immunohistochemistry respectively. The lung fibroblasts were isolated from mice and then co-cultured with breast cancer to identify the influence of cancer on the change of lung fibroblasts in PMN.

**Results:**

We demonstrated changes in the lipid profile and several lipid metabolism genes in the lungs of breast cancer-bearing MMTV-PyVT mice before cancer spreading. The expression of ACACA (acetyl-CoA carboxylase α) was downregulated in the lung fibroblasts, which contributed to changes in acetylation of protein’s lysine residues and the synthesis of fatty acid. The downregulation of ACACA in lung fibroblasts triggered a senescent and inflammatory phenotypic shift of lung fibroblasts in both in vivo and in vitro models. The senescence-associated secretory phenotype of lung fibroblasts enabled the recruitment of immunosuppressive granulocytic myeloid-derived suppressor cells into the lungs through the production of CXCL1 in the lungs. Knock-in of ACACA prevented lung metastasis in the MMTV-PyVT mouse model, further supporting that ACACA was involved in the remodeling of the lung PMN.

**Conclusions:**

Taken together, these data revealed a mechanism by which ACACA downregulation directed the formation of an immunosuppressive lung PMN in breast cancer.

**Supplementary Information:**

The online version contains supplementary material available at 10.1007/s13402-022-00767-5.

## Background

Breast cancer is a major global public health issue with a high incidence and mortality rate in women [1, 2]. Although there have been significant advances in conventional breast cancer treatment involving surgery, radiation, chemotherapy, immunotherapy and targeted therapy, many patients develop resistance to these therapies during the course of disease, eventually resulting in cancer recurrence and metastasis [3]. The high mortality rate of breast cancer is strongly associated with the development of metastasis. Breast cancer is associated with a high incidence of lung metastasis with a frequency ranging from 21 to 36% [4]. Therefore, it is vital to develop new therapies for the treatment of breast cancer.

The establishment of metastatic disease involves complex steps, including the detachment of cancer cells from the primary tumor, intravasation and circulation in the blood stream, followed by extravasation at secondary organs, adaptation to the new environment, and subsequently the development of metastases [5]. Over the last few years, the formation of pre-metastatic niche (PMN) has been shown to play a vital role in metastasis [6, 7]. The formation of a PMN provides an opportunity for disseminated cancer cells to efficiently adapt and survive in the metastatic niche. Tumor-derived factors are considered to be regulators of the initiation and remodeling of the PMN, which involves distant interactions between the primary tumor, PMN stromal components, and tumor mobilized bone marrow-derived cells (BMDCs) or immune cells [6, 8, 9]. However, it is still unknown how the immune cells are recruited to the PMN and how important this is to metastasis. A deep understanding of the formation and characteristic of PMN formation is required to develop new therapeutic strategies to treat patients with breast cancer.

Cellular metabolism consists of a complex network of enzymes, substrates and signaling pathways involving in multiple, tightly controlled, cellular processes that maintain homeostasis and adapt to normal physiological changes, such as those produced by the circadian rhythm, nutrient intake, and physical activity [10, 11]. Cancer cells reprogram their metabolism, which allows them to adapt to high growth and cope with the stress produced by hypoxia and nutrient deficiency in the microenvironment during cancer progression [11, 12]. The reprogramming of cancer metabolism also allows the cancer cells to uptake nutrients from the tumor microenvironment (TME), which then weakens immune cells by limiting the availability of nutrients [13]. In addition, increasing evidence has shown that cancer cells can induce neighboring stromal cells to produce proteins and metabolites to promote cancer development, progression, and drug resistance [14, 15]. A reverse Warburg effect of cancer-associated fibroblasts (CAFs) has been reported to facilitate biogenesis in tumor cells by increasing glycolysis and producing high-energy nutrients [16]. However, whether metabolic reprogramming is also involved in the formation of a PMN created by cancer cells has not previously been investigated. Therefore, the aim of this study was to investigate the role of metabolism reprogramming in the remodeling of the lung PMN. The results showed that primary breast cancer induced an inflammatory senescent phenotype of lung fibroblasts leading to immature immune cell infiltration before cancer spreading. Therefore, metabolic reprogramming not only plays a critical role in cancer-associated surrounding tissue, but plays an important role in shaping PMN metastatic progression.

## Methods

### Cell culture

MCF7 (HTB-22, ATCC) and Hs 578T (HTB-126, ATCC) cells were cultured in Minimum Essential Medium (MEM) and Dulbecco's Modified Eagle's Medium (DMEM) supplemented with 10% FBS. Normal human lung fibroblasts (NHLF) (38,128, Millipore) were cultured in Fibroblast Basal Medium (FBM) (C-3131, Lonza) containing FGM-2 Fibroblast Growth Medium-2 SingleQuots™ Supplements and Growth Factors (CC-4126, Lonza). Mouse lung epithelial cell line MLE-12 was obtained from ATCC and cultured in Dulbecco's medium/Ham's F12, medium containing 2% FBS, insulin (0.005 mg/mL), transferrin (0.01 mg/mL), Sodium selenite (30 nM), Hydrocortisone (10 nM), β-estradiol (10 nM), HEPES (10 mM) and L-glutamine (2 mM). Cells were routinely validated by short tandem repeat (STR) genotyping and periodically tested to ensure the absence of mycoplasma contamination. All cell lines were maintained at 37℃ in a humidified 5% CO_2_ atmosphere.

### Mouse models

Two different genetically engineered mouse models were used in this study: FVB/N-Tg (MMTV-PyVT) 634 Mul/J (also known as PyVT), and doxycycline-inducible (rtTA) ACACA expression mice (MMTV-PyVT/ACACA). The MMTV-PyVT mice were obtained from Jackson Laboratory, and the MMTV-PyVT/ACACA model was generated by crossing the MMTV-PyVT mice with the doxycycline (DOX)-inducible ACACA knock-in mouse line (National laboratory animal center narlabs, Taipei, Taiwan**)**. Briefly, ACACA transgene expression was controlled by a doxycycline-inducible promoter (Tet-On 3G Inducible Expression System (with emGFP). The ACACA expression was specifically induced from puberty in female mice at 9^th^ week through doxycycline-containing food (TD.01306, Envigo). At 12^th^ week, MMTV-PyVT and MMTV-PyVT/ACACA mice were euthanized, and the lungs were collected for H&E staining (n = 6 for each groups) (see below). The lungs of wild-type FVB and MMTV-PyVT mice at 9^th^ week were collected for microarray analysis. Genotyping was performed by PCR of DNA extracted from mice tail tips using three sets of primers: Transgene forward: GGAAGCAAGTACTTCACAAGGG and transgene reverse: GGAAAG TCACTAGGAGCAGGG, and internal control (forward: CAAATGTTG CTTGTCTGGTG; reverse: GTCAGTCGAGTGCACAGTTT). The touchdown PCR conditions were 94℃ for 10 s of initial denaturation, followed by 10 cycles of 94℃ for 10 s, 65–55℃ for 30 s and 72℃ for 1 min and 10 s, and then 31 cycles of 94℃ for 10 s, 55℃ for 30 s, and 72℃ for 1 min and 10 s; the final extension was at 72℃ for 3 min. All protocols related to animal use and treatment were evaluated and approved by the Animal Care and Use Committee of Kaohsiung Medical University, and all animals were treated in accordance with the National Laboratory for Experimental Animals guidelines.

### Microarray analysis of the mice lungs

The lungs of wild-type FVB and MMTV-PyVT mice (*n* = 3 each group) at 9^th^ week were collected, and the RNA was extracted using TRIZOL reagent. Total RNA (0.2 μg) was amplified using an Agilent Low Input Quick-Amp Labeling kit (Agilent Technologies, USA) and labeled with Cy3 (CyDye, Agilent Technologies, USA) during the in vitro transcription process. Cy3-labled cRNA (0.6 μg) was fragmented to an average size of about 50–100 nucleotides, and correspondingly fragmented labeled cRNA was hybridized using an SurePrint G3 Mouse GE 8 × 60 K Microarray (Agilent Technologies, USA). The microarrays were scanned with an Agilent microarray scanner (Agilent Technologies, USA) at 535 nm for Cy3. Scanned images were analyzed using Feature Extraction 10.7.3.1 software (Agilent Technologies, USA), image analysis and normalization software which is used to quantify signal and background intensity for each feature. Raw signal data were normalized by quantile normalization to determine the differentially expressed genes (DE genes).

### Lipid profile of lungs

Lipidomics was performed with lungs of wild-type and MMTV-PyVT mice (*n* = 10 each group) using an Ultra High-Performance Liquid Tandem Chromatography Quadrupole Time of Flight Mass Spectrometry (UHPLC-QTOFMS) (Biotree, Shanghai). Briefly, equal weight of lungs were homogenized with 400 ml H_2_O, and then mixture with 960 ml MTBE/MeOH (5:1) solution (MTBE: CNW Technologies, 1643–04-4; Methanol: CNW Technologies, 67–56-1) containing 5 mL 10 ppm d7-PE (15:0/ 18:1) (Avanti), 1.5 mL 100 ppm d7-LPC (18:1) (Avanti), 1.5 mL 100 ppm d7-TG (15:0/18:1/15:0) (Avanti). After centrifuge, the supernatant were dried and then dissolved in 100 ml DCM / MeOH (1:1, v/v) (DCM: Merck, 75–09-2) solution. Sixty ml of each sample were assessed by UHPLC (1290, Agilent Technologies)-QTOF (AB Sciex), which equipped with a Phenomen Kinetex 1.7u C18 100A Column (100 × 2.1 mm), coupled to a Triple TOF 6600 (Q-TOF, AB Sciex). The mobile phase consisted of A: 10 mM HCOONH_4_, 40% H_2_O and 60% ACN, B: 10 mM HCOONH_4_, 10% CAN and 90% IPA was carried with elution gradient as follows: 0 min, 40% B; 12 min,100% B; 13.5 min, 100% B; 13.7 min, 40% B; 18 min, 40% B, which was delivered at 0.3 mL/min. The injection volume was POS: 0.5 mL, NEG: 5 mL. The Triple TOF mass spectrometer was used to acquire MS/MS spectra on an information-dependent basis (IDB) during an LC/MS experiment. In this mode, the acquisition software (Analyst TF 1.7, AB Sciex) continuously calculated the full scan survey MS data as it collects and triggers the acquisition of MS/MS spectra depending on preselected criteria. In each cycle, 12 precursor ions whose intensity greater than 100 were chosen for fragmentation at collision energy (CE) of 35 V (15 MS/MS events with product ion accumulation time of 50 ms each). ESI source conditions were set as following: Ion source gas 1 as 60, Ion source gas 2 as 60, Curtain gas as 30, source temperature 550℃, Ion Spray Voltage Floating (ISVF) 5500 V or 4500 V in positive or negative modes, respectively.

### Quantitative real-time polymerase chain reaction (qRT-PCR)

The qRT-PCR was performed using the 2 ^− ΔΔCt^ method. RNA was converted to cDNA using a PrimeScriptTM RT Reagent Kit (RR037A, TaKaRa), and 50 ng cDNA per reaction were used with Fast SYBR Green Master Mix (4,385,612, Applied Biosystems), followed by reactions using a QuantStudio 3 machine (Applied Biosystems). GAPDH was used as the internal control gene to give the tested genes a relative fold change using the 2 ^− ΔΔCt^ method. The qRT-PCR primers are listed in sTable 1.

### Cell isolation from the lungs of mice

For fibroblasts, resected lungs were chopped into pieces, followed by incubation with collagenase cocktail (250 ug/ml collagenase I, II, and IV in DMEM) on a rotator for ∼1–2 h. To isolate lung fibroblasts, immune cells (CD45^+^), epithelial cells (EPCAM^+^) and endothelial cells (CD31^+^) were excluded using anti-CD45, anti-EPCAM and anti-CD31 microbeads, and the cells were seeded in a culture dish. The next day, the monolayer was washed several times and fresh media was added until the cells were ready for further processing. Granulocytic myeloid-derived suppressor cells **(**G**-** MDSCs, (CD45^+^CD11b^+^Gr-1^+^), monocytic MDSC (M-MDSC, CD45^+^CD11b^+^Gr-1^−^) and Regulatory T cells (Treg) (CD45^+^CD4^+^CD25^+^) from pulmonary suspensions were separated and isolated using a magnetic cell separation system (MACS) (Miltenyi Biotec, Bergish Gladbach, Germany).

### siRNA transfection

The ACACA expression in lung fibroblasts was downregulated by the RNAi-mediated inhibition of RNA expression, using a mixture of four siRNAs for each target gene (Acell; Dharmacon, Lafayette, CO, USA) as previously described. A non-targeting siRNA pool (Dharmacon) was used as the control. In brief, exponentially growing cells were seeded in regular growth medium without antibiotics at 50% of confluence, and the cells were transfected with siRNA according to the manufacturer’s instructions. The cells were then incubated to verify the knockdown efficiency after 48 h before the experiments.

### Immunoblot experiments

Total protein of cell lysates was extracted using a radioimmunoprecipitation assay buffer with 1% phosphatase inhibitor cocktail 2 (catalog no. P5726, Sigma-Aldrich) following the manufacturer’s recommendations. The concentration of protein sample was determined using a bicinchoninic acid (BCA) assay kit (Thermo Fisher Scientific). And protein was then denatured at 95℃ for 5 min. An equal amount of protein (30 μg) was loaded onto 10% SDS–polyacrylamide gel for electrophoresis and transferred to a polyvinylidene difluoride membrane. Membranes were exposed to the antibodies as listed in sTable 2. They were then developed using an enhanced chemiluminescence Substrate kit (EMD Millipore), imaged using AlphaImager, and the levels of target proteins were normalized to those of GAPDH based on quantitation using AlphaEaseFC (Alpha Innotech, San Leandro, CA, USA).

### Immunohistochemistry (IHC)

Lungs of the mice were analyzed using IHC with p53 rabbit polyclonal antibody (ab31333, Abcam), ACTA2 (α-SMA) rabbit polyclonal antibody (orb234994, Biorbyt) and ACACA (ACC1) rabbit polyclonal antibody (GTX132081, GeneTax). Data were analyzed with TissueFAXS **(**TissueGnostics, Vienna, Austria) based on the IHC staining slides.

### Senescence-associated β-galactosidase (SA-β-GAL) staining for senescent cells

Human breast cancer cell lines and mice primary breast tumor cells were seeded at 2 × 10^4^ cells in 24-well hanging 3.0 μm inserts (MCSP24H48, Millipore) and co-cultured with 1 × 10^4^ cells of lung fibroblasts in the wells of a 24-well plate. The inserts and fibroblast media were removed after 120 h of co-culture. The fibroblasts were then fixed by 10% formaldehyde (3933, J.T.Baker) and stained by 500 μL solution (pH 6.0) from a Senescence Cells Histochemical Staining Kit (CS0030-1KT, Sigma) for 16 h incubation at a CO_2_-free, 37℃ condition. Each experiment was performed three times independently, and exemplary bright field 10X micrographs were taken using a microscope.

### Measurement of malonyl-CoA and acetyl-CoA

The levels of malonyl-CoA and acetyl-CoA in lung fibroblasts were determined using a malonyl-CoA ELISA kit (CSB-E12896, CUSABIO) and acetyl-CoA ELISA kit (CSB-E12936, CUSABIO). The cell lysates were normalized by total protein using a bicinchoninic acid protein assay.

### Cell migration

Cell migration assays were conducted using the 3 μm inserts (EMD Millipore). Mouse lung fibroblasts (1 × 10^4^ cells/well) were co-cultured with mouse primary breast cancer (2 × 10^4^ cells/well) for 120 h, and G-MDSCs (1 × 10^6^ cells/well) were seeded onto inserts for 6 h. Migratory or invade cells were counted using a fluorescence microscope. For recombinant (rm) mouse CXCL1 protein analysis, G-MDSC was seeded onto inserts and rm CXCL1 protein (20 ng/mL) was added into the bottom as a chemoattractant. After 6 h, the migratory G-MDSC was counted. For blocking study, the IgG (control) or anti-CXCL1 antibodies (2 μg/mL) were added to the fibroblast co-cultured primary breast cancer to act as the neutralizing agents.

### Cell proliferation

Cell proliferation of breast cancer cells were assessed by PreMix WST-1 Cell Proliferation Assay System (Cat # MK400, -Bio, Tokyo, Japan). Breast cancer induced senescent fibroblasts were washed by PBS and cultured in fresh medium for 24 h, the supernatants were collected to act as condition medium (CM). Breast cancer cells were treated with CM (50%) of senescent fibroblasts for 48 h. Each experiment was performed in three wells and was repeated independently at least three times.

### Statistical analysis

Data were analyzed using Prism 9.0.0 (GraphPad Software) with either the unpaired t test or one-way ANOVA when more than two groups were compared. The presented results are representative of three independent experiments with similar results. All data are presented as mean ± S.D., and a p value less than 0.05 was considered to be statistically significant.

## Results

### Lipid metabolism-related genes were downregulated in the lung PMN

**Fig. 1:**
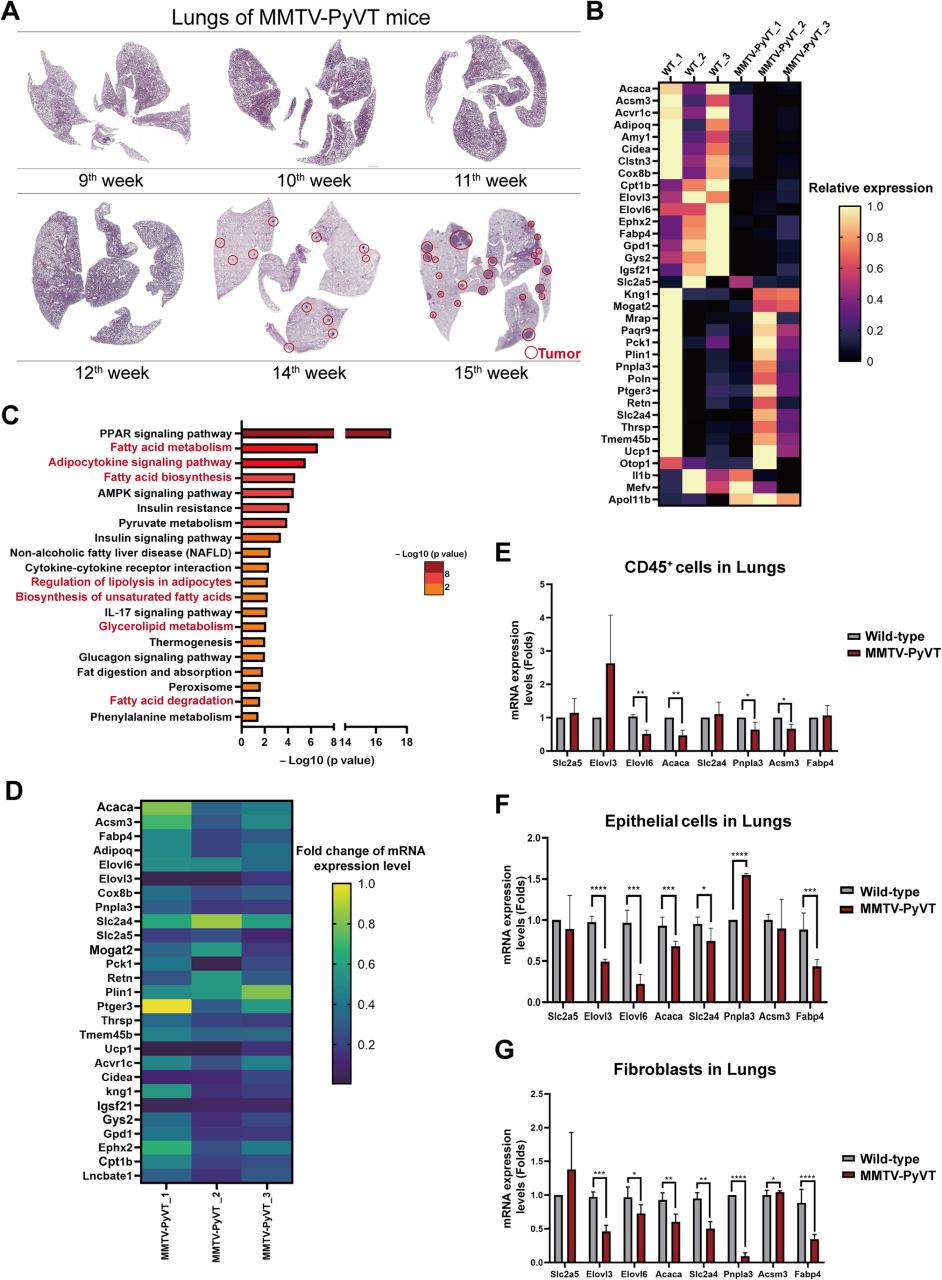
Enzyme lipid metabolism was altered in the lung PMN of mice. (**A**) The H&E staining of the lungs of MMTV-PyVT mice at different weeks of age (9^th^ to 15^th^ week). (**B**) Heat-map of upregulated and downregulated genes of lungs of MMTV-PyVT mice. The lung of wild-type and breast cancer-bearing MMTV-PyVT mice at 9^th^ week (*n* = 3 in each group) were collected, and the gene profile was established by microarray. (**C**) The KEGG pathway analysis of transcriptomes of the lungs of MMTV-PyVT mice. (**D**) The levels of lipid metabolism-related genes in the lung tissue as determined by qRT-PCR. The expressions of lipid metabolism-related genes in CD45^+^ cells (**E**), lung epithelial cells (**F**), and lung fibroblasts (**G**). The lungs of wild-type and breast cancer-bearing MMTV-PyVT mice at 9^th^ week (*n* = 3 in each group) were collected, and the gene profile was established by microarray or qRT-PCR. All cells were isolated from the lungs of mice using microbeads. All results are representative of at least three independent experiments. Graphs show mean ± S.D. *, *P* < 0.05; **, *P* < 0.01; ***, *P* < 0.001; ****, *P* < 0.0001

To determine which factors were involved in lung PMN formation, we assessed the lungs of breast cancer-bearing MMTV-PyVT mice from 9^th^ to 15^th^ week. Before 12^th^ week, no tumor nodules were found in the lungs of the mice (Fig. 1A and sFig. 1). Microarray analysis showed that there were 32 downregulated and 3 upregulated genes in the lungs of the breast cancer-bearing MMTV-PyVT mice (Fig. 1B). Pathway analysis showed that several lipid-metabolism pathways were affected in the lungs of the MMTV-PyVT mice, including “Fatty acid metabolism”, “Adipocytokine signaling pathway”, “Fatty acid biosynthesis”, “Regulation of lipolysis in adipocytes”, “Biosynthesis of unsaturated fatty acids”, “Glycerolipid metabolism”, and “Fatty acid degradation” (Fig. 1C, sFig. 2A and 2B). Among 32 downregulated genes, 10 genes of the regulated genes (*Acaca, Acsm3, Adipoq, Elovl3, Elovl6, Fabp4, Pnpla3, Slc2A4, Slc2A5, Cox8b*) were related to lipid metabolism, and qRT-PCR analysis also verified that they were decreased in the lungs of the MMTV-PyVT mice before lung metastasis (Fig. 1D).

To further investigate changes in the lipid metabolism genes in different types of cells, we isolated immune cells (CD45^+^ cells), epithelial cells (EPCAM^+^), and fibroblasts from the lungs of wild-type and MMTV-PyVT mice. The results revealed that *Acaca, Acsm3, Pnpla3* and *Elovl6* were downregulated in CD45^+^ cells, while *Acaca, Slc2a4, Fabp4, Elovl3 and Elovl6* were reduced in epithelial cells (Fig. 1E and F). *Acaca*, *Elovl3, Elovl6, Fabp4, Slc2a4 and pnpla3* were downregulated in fibroblasts. Co-culture of primary breast cancer (PBC) cells isolated form mice with lung epithelial cell MLE-12 also reduced the expression of *Elovl6, Fabp4, Pnpla3, Slc2a5, Adipoq and Cox8b *(sFig. 2C). In addition, PBC cells decreased the expression of *Acaca* and *Pnpla3* in lung fibroblasts isolated from lungs of wild-type mice in a co-culture system (sFig. 2D)*.*

### Changes in lipidomics of the lung PMN in mice

**Fig. 2:**
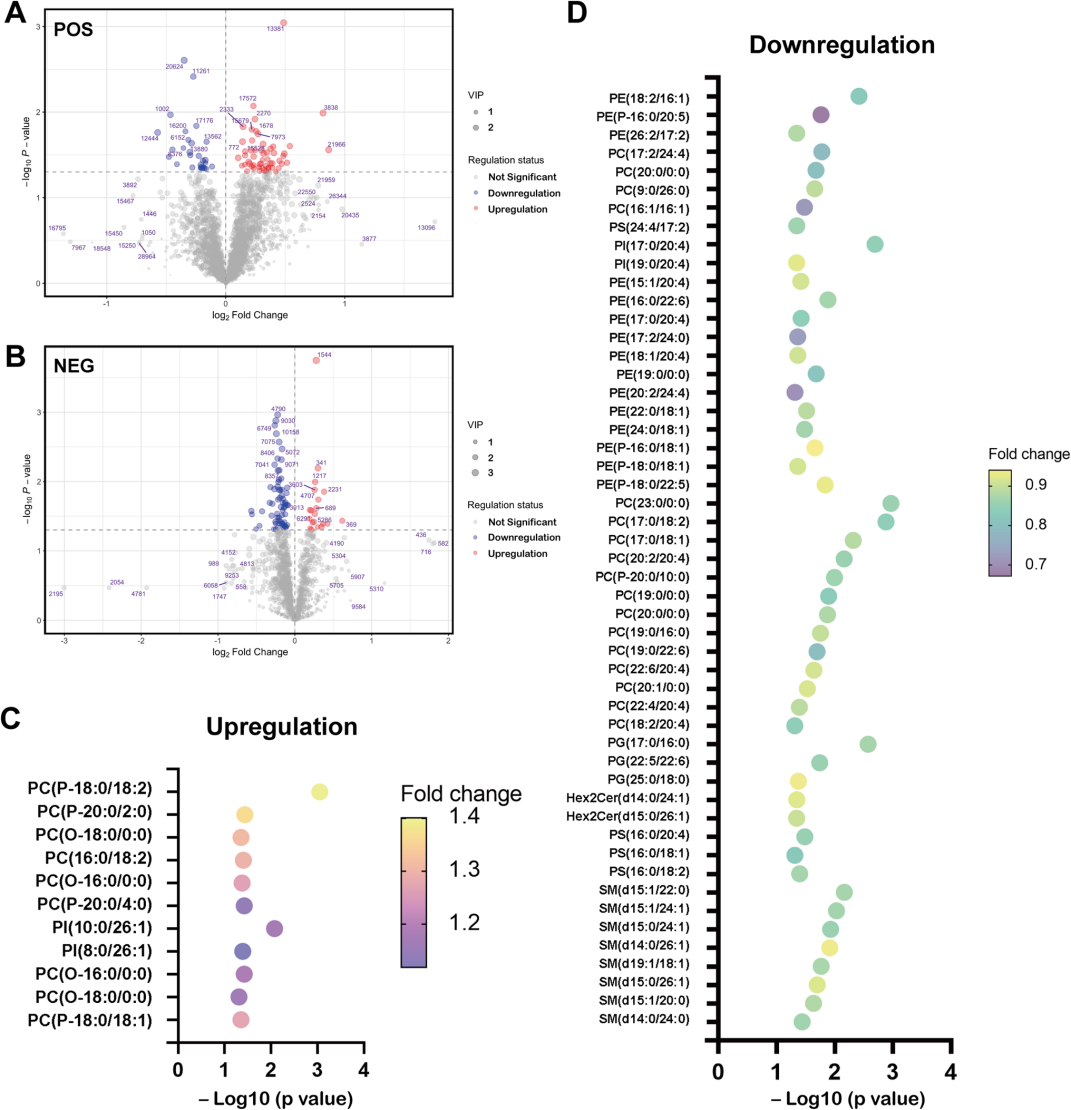
Lipidomics of the mice lungs. (**A**) Volcano plot of positive ion mode of fatty acid. (**B**) Volcano plot of negative ion mode of fatty acids. (**C**) The lipids upregulated in the lungs of MMTV-PyVT mice. (D) DE lipids were downregulated in the lungs of MMTV-PyVT mice. The lungs of mice at 10^th^ week (n = 10 in each group) were collected, and the fatty acids were assessed using liquid chromatography-quadrupole time-of-flight mass spectrometry

We then performed lipidomics and analyzed the lungs of wild-type and MMTV-PyVT mice. The lipid profiles of mice lung tissue, including 18 lipid species, 7 in positive ion mode and 11 lipid species in negative ion mode, were selected by nontargeted lipidomics from a total of 20 lung samples. There were 7 carnitine, 15 cholesteryl ester (CE), 43 ceramide (Cer), 15 cardiolipin (CL), 20 diacylglycerol (DG), 13 dihexosylceramide (Hex2Cer), 13 hexosylceramide (HexCer), 4 2-monoglyceride (MG), 53 phosphatidic acid (PA), 289 phosphatidylcholine (PC), 196 phosphoethanolamine (PE), 112 phosphatidylglycerol (PG), 45 phosphatidylinositol (PI), 86 phosphatidylserine (PS), 69 sphingomyelin (SM), 9 sphingosine (SP), and 128 triglyceride (TG) (Fig. 2A and B). Typical total ion chromatography (TIC) of the lipid profiles is provided in sFig. 3A and 3B. Fifty-three fatty acids, including PC, PE, PS, SM, Hex2Cer were shown to be significantly decreased, and 11 fatty acids were shown to be significantly increased in the lungs of the MMTV-PyVT mice (Fig. 2C and D). These data suggested that the lipidomics of lung in the cancer-bearing mice were changed before cancer metastasis.

### ACACA was downregulated in the fibroblasts of the lung PMN

**Fig. 3:**
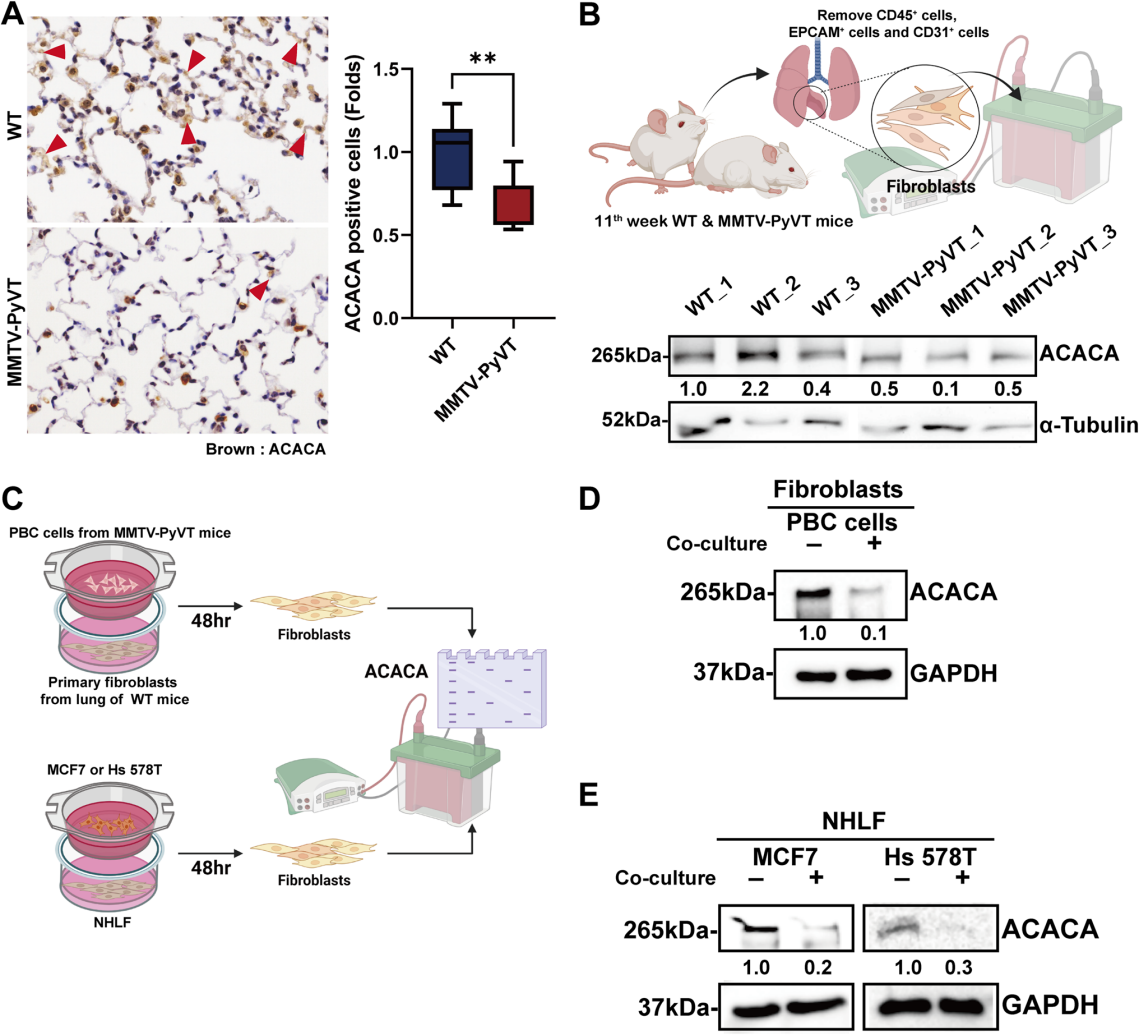
Downregulation of ACACA in the lung fibroblasts of cancer-bearing MMTV-PyVT mice. (**A**) The level of ACACA in the lungs of wild-type (WT) and MMTV-PyVT mice, as determined by IHC at 10^th^ week (n = 10 in each group). (**B**) The ACACA level in the lung fibroblasts isolated from the WT and MMTV-PyVT mice at 11^th^ week (n = 3 in each group), as determined by immunoblot. (**C**) The schematic diagram of experiment. (**D**) Mouse primary breast cancer (PBC) cells decreased ACACA expression in lung fibroblasts in a co-culture system. Lung fibroblasts were isolated from the lungs of wild-type mice, and mouse PBC cells were isolated from primary mammary site of MMTV-PyVT mice. The fibroblasts and mouse PBC cells were co-cultured in a transwell system for 48 h, and the expression of ACACA in fibroblasts was assessed by immunoblot. (**E**) Human breast cancer cells decreased the ACACA expression in fibroblasts. Normal human lung fibroblast (NHLF) and human breast cancer cell lines (MCF7 and Hs 578T) were co-cultured in a transwell system for 48 h, and the expression of ACACA in fibroblasts was determined by immunoblot. Graphs show mean ± S.D. **, *P* < 0.01

As ACACA is the initiator and rate-limiting enzyme of fatty acid biosynthesis [17], and as it was downregulated in lung fibroblasts, the most common cell type in tissue, we assumed that ACACA may play an important role in PMN formation in the lungs. IHC staining revealed that the level of ACACA was downregulated in the lungs of the breast cancer-bearing MMTV-PyVT mice (Fig. 3A). Fibroblasts isolated from the lungs of these mice also expressed lower levels of ACACA at protein levels compared with the fibroblasts from wild-type mice (Fig. 3A). A co-culture system also showed that PBC cells isolated from primary sites of the mice also had lower expressions of ACACA in the lung fibroblasts isolated from the wild-type mice (Fig. 3C and D). Similarly, in human breast cancer cells, including MCF7 and Hs 578T, caused a reduced expressions of ACACA were found in lung fibroblasts in a co-culture model (Fig. 3C and E). These results indicated that primary breast cancer reduced the expression of ACACA in lung fibroblasts via a distant interaction.

### The synthesis of fatty acid and goblet acetylation of lysine residue was changed in lung fibroblasts

**Fig. 4:**
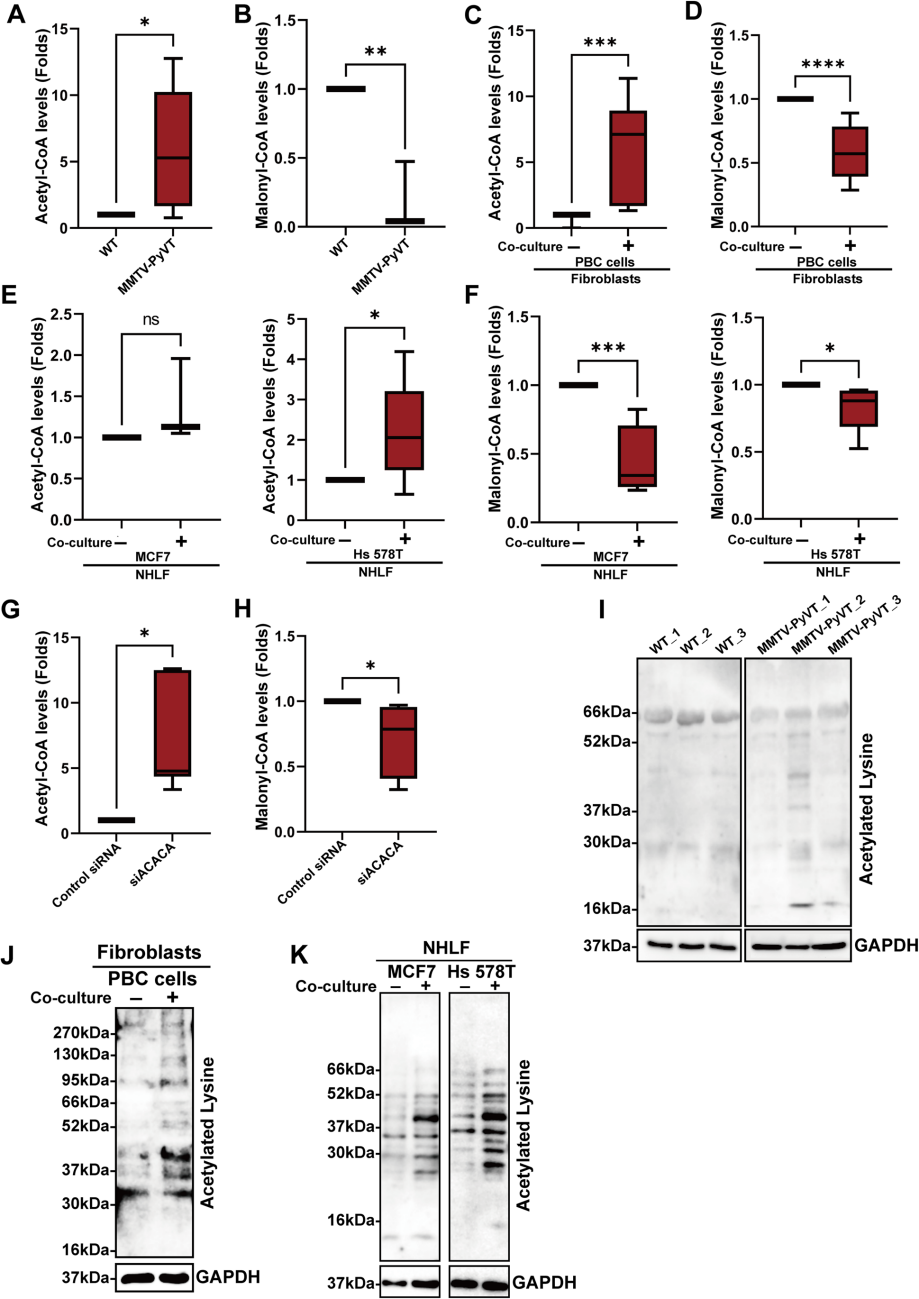
The activity of ACACA was decreased in the lung fibroblasts of cancer-bearing MMTV-PyVT mice. The activity of ACACA was decreased in the lung fibroblasts, as evaluated by the levels of acetyl-CoA (A) and malonyl-CoA (B) in situ at 12^th^ week (n = 6 in each group). The elevated acetyl-CoA (C) and reduced malonyl-CoA (D) were found in mouse lung fibroblasts co-cultured with PBC cells. The lung fibroblasts were isolated from wild-type mice and then co-cultured with mice PBC cells for 48 h. The levels of acetyl-CoA and malonyl-CoA were determined by ELISA (n = 5 in each group). Human breast cancer cells also decreased ACACA activity of lung fibroblasts (NHLF), as determined by the levels of acetyl-CoA (E) and malonyl-CoA (F). Normal human lung fibroblasts and human breast cancer cell lines (MCF7 and Hs 578T) were co-cultured in a transwell system for 48 h, and the levels of acetyl-CoA and malonyl-CoA were determined by ELISA (n = 4 in each group). Knockdown of ACACA altered the metabolism of acetyl-CoA (G) and malonyl-CoA (H) (n = 4 in each group). The expression of NHLF was inhibited by siRNA delivery, and the expression of ACACA was assessed by Immunoblot. (I) The levels of lysine acetylation of total protein in the lung fibroblasts isolated from MMTV-PyVT and wild type mice at 12^th^ week (n = 3 in each group). (J) The levels of lysine acetylation of total protein in mouse lung fibroblasts co-cultured with mouse PBC. (K)The levels of lysine acetylation of total protein in the NHLF co-cultured with MCF7 (or Hs 578T) in a transwell system for 48 h, and the level of lysine acetylation in NHLF was evaluated by immunoblot. Graphs show mean ± S.D. *, *P* < 0.05; **, *P* < 0.01; ***, *P* < 0.001; ****, *P* < 0.0001

As ACACA is an enzyme which catalyzes the carboxylation of acetyl-CoA to malonyl-CoA [18], we evaluated the activity of ACACA by determining the concentrations of substrates and products. As shown in Fig. 4A and B, the cellular level of acetyl-CoA increased while the level of malonyl-CoA decreased in the fibroblasts isolated from the lungs of breast cancer-bearing MMTV-PyVT mice. Co-culture of lung fibroblasts with PBC cells increased the level of acetyl-CoA and decreased the level of malonyl-CoA (Fig. 4C and D). Similarly, co-culturing human lung fibroblasts with human breast cancer cells also with an elevated acetyl-CoA and reduced the level of malonyl-CoA (Fig. 4E and F). Knockdown of ACACA by siRNA also resulted in a reduction in product-to-substrate ratio in human lung fibroblasts (Fig. 4G and H), suggesting that ACACA activity was attenuated in lung fibroblasts by primary cancer.

Acetyl-CoA has been reported to contribute to lysine acetylation [19], and therefore we assessed the status of acetylation in various protein in lung fibroblasts. As shown in Fig. 4I, the increased acetylation of total protein at lysine residue was found in the fibroblasts isolated from MMTV-PyVT mice, compared with the fibroblasts obtained from wild type mice. The enhanced acetylation of total protein at lysine residue was found in mouse lung fibroblasts and human fibroblasts co-cultured with mouse and human breast cancer cells, respectively (Fig. 4J and K). These results suggested that breast cancer changes the acetylation-related modification of proteins in fibroblasts of lung PMN in breast cancer.

### Inhibition of ACACA induced senescence in lung fibroblasts

**Fig. 5:**
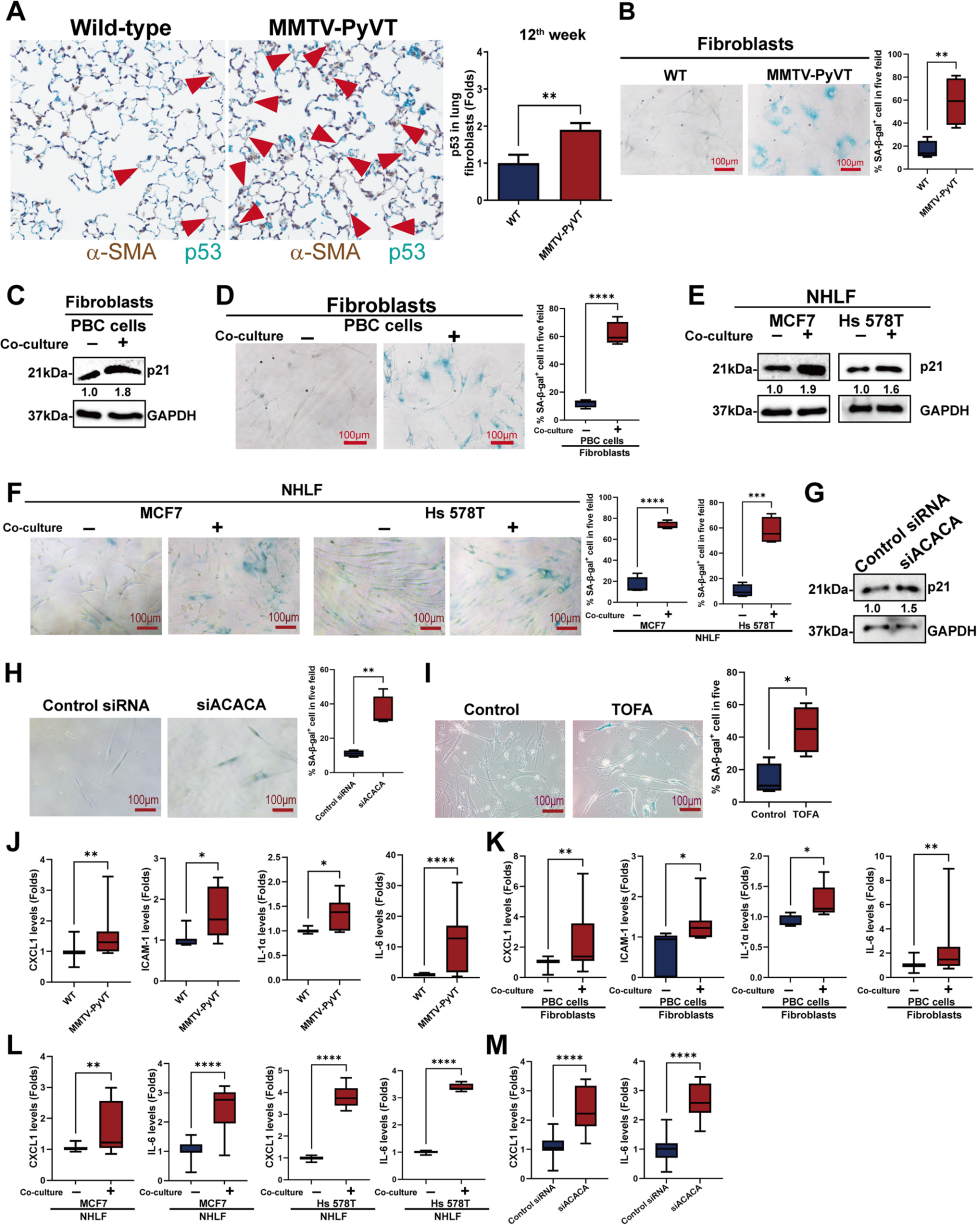
The senescence phenotype of ACACA-downregulated lung fibroblasts. (**A**) The senescent phenotype in lung PMN of mice. The p53 in the lung of mice at 12^th^ week stained by IHC. (**B**) β*-*galactosidase staining of lung fibroblasts isolated from MMTV-PyVT and wild type mice at 12^th^ week. Primary breast cancer (PBC) cells induced senescence in lung fibroblasts, as determined by p21 upregulation (**C**) and β*-*galactosidase staining (**D**) in a co-culture system. Human breast cancer cells induced senescence in human lung fibroblasts, as determined by p21 upregulation (**E**) and β*-*galactosidase staining (**F**). Normal human lung fibroblasts (NHLF) and human breast cancer cell lines (MCF7 and Hs 578T) were co-cultured in a transwell system for 120 h. The senescent phenotype was assessed by p21 expression and β*-*galactosidase staining, as determined by immunoblot. Inhibition of ACACA by siRNA transfection induced senescence in NHLF as assessed by p21 upregulation (**G**) and senescence-associated beta-galactosidase analysis (**H**). NHLF were transfected with ACACA siRNA for 120 h, and the cells was stained using cell senescence kits. The fibroblasts received a booster shot of ACACA Acell siRNA every 3 days. (**I**) An ACACA chemical inhibitor, TOFA, induced fibroblast senescence. NHLF was treated with TOFA (0.5 μg/ml) for 3 days. The phenotype was evaluated using cell senescence kits. (**J**) Senescence-related cytokines (CXCL1, IL-1α, IL-6 and ICAM-1) were upregulated in the lungs of breast cancer-bearing MMTV-PyVT mice (n = 16 in each group). The levels of various cytokines in the lung fibroblasts of mice were assessed using a Luminex system. The co-culture of lung fibroblasts with PBC cells (n = 12 in each group) (**K**) or human breast cancer cells (n = 4 in each group) (**L**) increased the expressions of various cytokines. Mouse lung fibroblasts or primary human fibroblasts were co-cultured with mice breast cancer cells or human cancer cell lines for 120 h. (**M**) ACACA inhibition by siRNA transfection increased the senescence*-*associated secretory phenotype in lung fibroblasts (n = 6 in each group). NHLF were transfected with ACACA siRNA for 120 h, and the levels of various cytokines were assessed using a Luminex system. Graphs show mean ± S.D. *, *P* < 0.05; **, *P* < 0.01; ***, *P* < 0.001; ****, *P* < 0.0001

A previous study reported that a reduction of ACACA in human primary fibroblasts promoted replicative senescence [20]. Senescence is a process following genotoxic stimuli and induces permanent cell cycle arrest with a loss of cellular functions. Senescent cells secrete several inflammatory cytokines, growth factors, proteases, and other factors, which collectively are indicated as the senescence-associated secretory phenotype (SASP) [21]. We evaluated whether breast cancer altered the phenotype of fibroblasts in the lung PMN. IHC results revealed that, compared with the lungs of wild-type mice, the senescence markers p53 was increased in the lungs of breast cancer-bearing MMTV-PyVT mice at 10^th^ to 12^th^ week but not at 9^th^ week (Fig. 5A, sFig. 4A to 4C). β-galactosidase staining of lung fibroblasts isolate from 12^th^ week MMTV-PyVT mice also supported the senescent phenotype induced by breast cancer (Fig. 5B). PBC cells induced the senescent phenotype in the lung fibroblasts isolated from wild-type mice, as supported by p21 upregulation and β-galactosidase staining in a co-culture system (Fig. 5C and D). Similarly, co-culture of human normal lung fibroblasts (NHLF) with human breast cancer cells MCF7 and Hs 578T also induced fibroblasts’ senescence, as represented by p21 and p27 upregulation, β-galactosidase staining and ki-67 loss, as well as pR6S increase [22] (Fig. 5E and F, sFig. 4D to 4F). In addition, inhibition of ACACA in lung fibroblasts also induced senescence through either siRNA-based silencing (Fig. 5G and H, sFig. 4G and 4H) or ACACA chemical inhibitor TOFA (Fig. 5I). These results suggest that breast cancer induces lung fibroblasts aging via an ACACA-dependent pathway.

Because senescent cells secrete various inflammatory factors, we next determined whether breast cancer induces lung fibroblasts to release various factors. We found that the expressions of CXCL1, ICAM-1, IL-1α and IL-6 were higher in the lung fibroblasts isolated from the lungs of MMTV-PyVT mice at 12^th^ week but not 9^th^ week compared with those in the wild-type mice (Fig. 5J, sFig. 4I and sTable 3). Co-culturing lung fibroblasts with PBC cells also increased the secretion of CXCL1, ICAM-1, IL-1α and IL-6 (Fig. 5K). Co-culturing lung fibroblasts with human breast cancer increased the secretion of CXCL1 and IL-6 (Fig. 5L). Knockdown of ACACA by siRNA transfection in lung fibroblasts also increased the expressions of CXCL1 and IL-6 (Fig. 5M).

### The lung microenvironment drove inflammation and G-MDSC infiltration by CXCL1

**Fig. 6:**
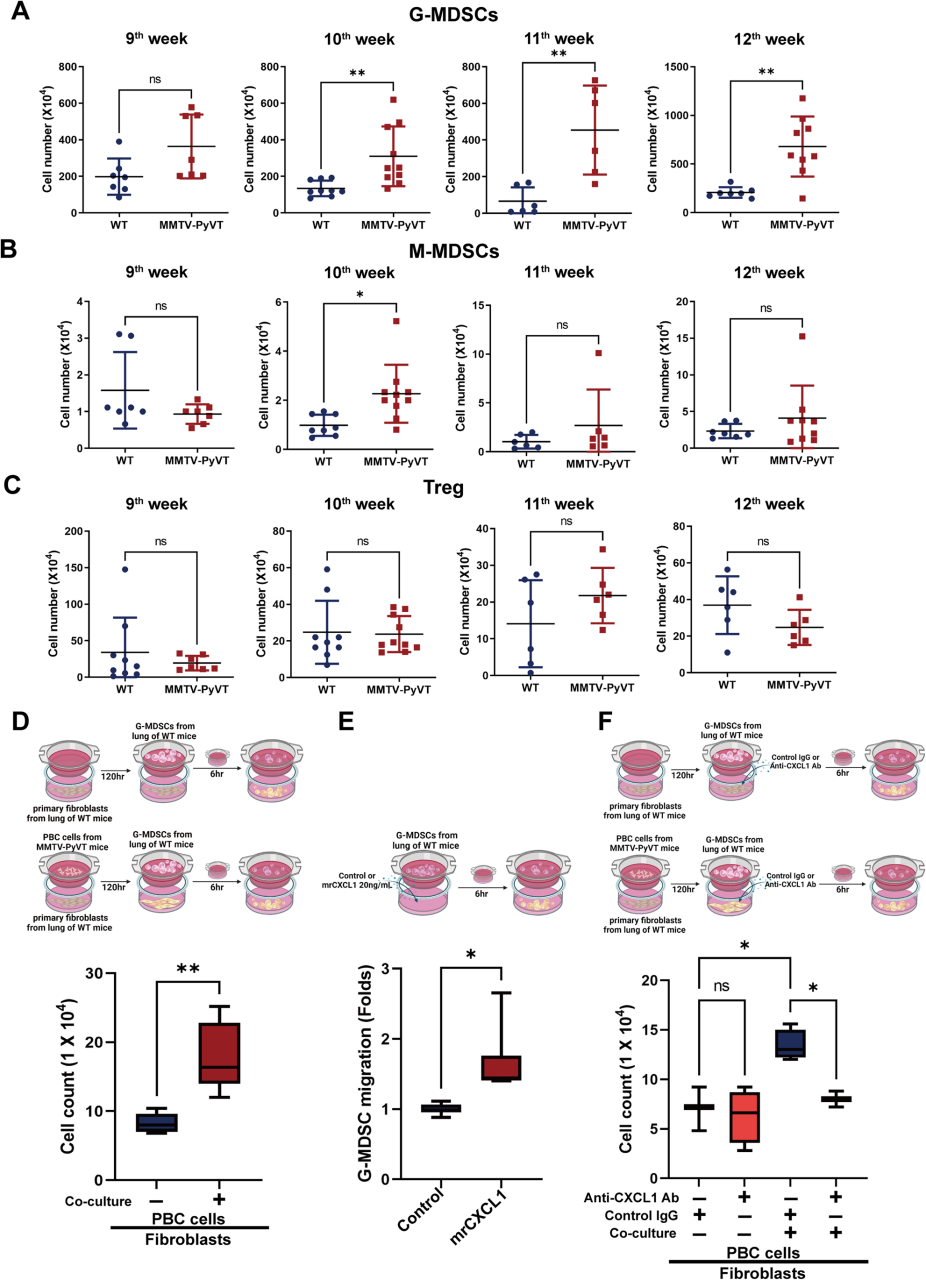
The infiltration of G-MDSC in the lungs of MMTV-PyVT mice. The cell number of (**A**) G-MDSCs, (**B**) M-MDSCs and (**C**) Treg in the lungs of mice from 9^th^ to 12^th^ week. G-MDSCs, M-MDSCs and Treg were isolated from the lungs of wild-type and MMTV-PyVT mice, and the number of cells was counted. (**D**) Senescent fibroblasts increased the migration of G-MDSCs. Mouse lung fibroblasts were co-cultured with mouse primary breast cancer (PBC) cells in a 24 well plate for 120 h. After removing the inserts, G-MDSCs were seeded in a new insert and then co-cultured with lung fibroblasts. The migration of G-MDSCs was evaluated after 6 h migration. (**E**) mrCXCL1 (20 ng/mL) increased the migration of G-MDSCs. (**F**) Blockade of CXCL1 decreased the effect of senescent fibroblasts in the chemoattraction of G-MDSCs. G-MDSCs were isolated from MMTV-PyVT mice and seeded in the inserts, which were hanged on the wells of the plate for 6 h. Graphs show mean ± S.D. *, *P* < 0.05; **, *P* < 0.01; ns, non-significant

Similar to the microenvironment surrounding the primary tumor, chronic inflammatory mediators can induce the infiltration of immunosuppressive cells. Therefore, we evaluate the recruitment of G-MDSCs, M-MDSCs and Treg cells in the lung PMN of wild-type and cancer-bearing MMTV-PyVT mice. As shown in Fig. 6A, G-MDSCs accumulated in the lungs of MMTV-PyVT mice at 10^th^ to 12^th^ week, but not at 9^th^ week, suggesting G-MDSCs infiltration was related to fibroblast senescence-associated inflammatory cytokine secretion. In contrast to G-MDSCs, the cell number of M-MDSCs or Treg cells did not change in the lungs of cancer-bearing MMTV-PyVT mic from 9 to 12^th^ week (Fig. 6B and C).

To determine whether fibroblast senescence was required for G-MDSC infiltration, we performed a transwell chamber assay. G-MDSCs were plated in the inserts and mouse lung fibroblasts, in which the senescent phenotype induced by PBC cells were seeded in the bottom. The results showed that senescent fibroblasts increased the migration of G-MDSCs (Fig. 6D). In addition, recombinant mouse CXCL1 protein increased the migration of G-MDSCs (Fig. 6E), whereas the enhanced G-MDSC migration induced by senescent fibroblasts was prevented by anti-CXCL1 antibodies (Fig. 6F). These results suggest that senescent fibroblast-secreted CXCL1 is critical for the infiltration of G-MDSCs.

To evaluate the role of senescent fibroblasts on the cancer behaviors, we assessed the CMs of senescent fibroblasts on cancer proliferation and migratory ability. The results showed that the CMs of MCF7 and Hs 578T-derived senescent fibroblasts increased the cell proliferation and migratory ability in MCF7 and Hs 578T cells, respectively (sFig. 5A and B), suggesting senescent fibroblasts have pro-metastatic feature.

### Restoration of ACACA prevented lung metastasis

**Fig. 7:**
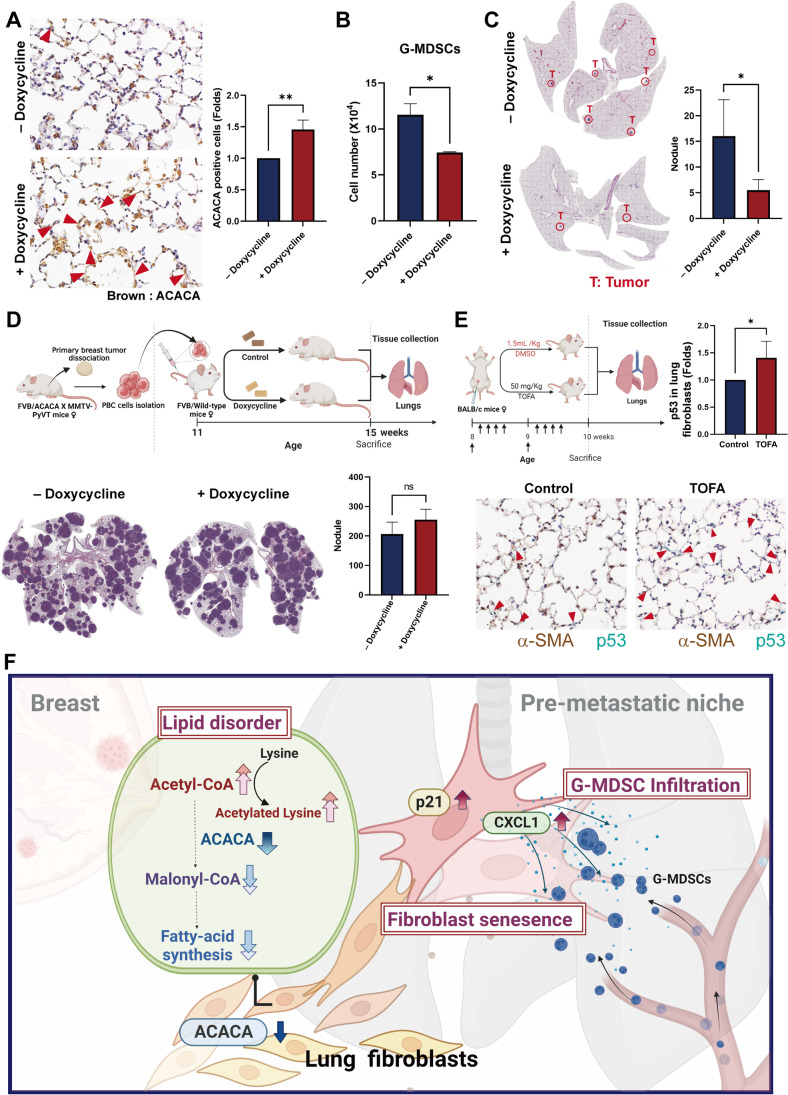
Overexpression of ACACA decreased lung metastasis in vivo. (A) The overexpression of ACACA in lungs of mice at 15^th^ week. (B) The recruitment of G-MDSCs in the lung of ACACA knock-in transgenic mice at 15^th^ week. (C) The tumor nodules in the lungs of ACACA knock-in transgenic mice at 15^th^ week (n = 4 in each group). (D) Overexpression of ACACA in breast cancer cells did not enhance lung metastasis in vivo (n = 4 in each group). (E) TOFA reduced fibroblast senescence in the lungs of mice. Mice were randomly divided into 2 groups: untreated (control) and TOFA (50 mg/kg) treated for 14 days. (F) The schematic diagram of our study. Primary breast cancer alters lipid metabolism of lung fibroblasts by ACACA downregulation. Lung fibroblasts convert to a senescent phenotype, which increases the recruitment of G-MDSCs by CXCL1 production. All results are representative of at least three independent experiments, and each value is the mean ± S.D. of three results; *P < 0.05

To evaluate the role of ACACA in the lung metastasis of breast cancer, we generated a doxycycline-inducible ACACA knock-in model (MMTV-PyVT/ACACA) (Fig. 7A). The infiltration of G-MDSCs, but not M-MDSCs and Treg, decreased in MMTV-PyVT/ACACA mice (Fig. 7B, sFig. 6A and 6B). Consistent with previous data, specific induction of ACACA in the lung fibroblasts of the PMN decreased lung metastasis in the ACACA knock-in mouse model (Fig. 7C). Interestingly, our results showed that the metastatic ability of ACACA-overexpression PBC cells did not change in a tail vein implantation model, compared with the PBC cells without doxycycline induction, suggesting that ACACA overexpression did not influence the spreading capacity of cancer cells (Fig. 7D). This finding eliminated the possibility that reduced lung metastasis in ACACA-knock-in mice was due to an increase in the metastatic ability of ACACA-overexpressed breast cancer cells. Similar to the transgenic mice, the ACACA inhibitor TOFA also decreased lung senescence of lung fibroblasts in a mouse model (Fig. 7E). Taken together, these results suggest that ACACA inhibition in the lung fibroblasts of the PMN contributes to metastasis of breast cancer.

## Discussion

Increasing evidence has indicated that an abnormal lipid metabolism level contributes to the progression of malignancy [23]. The PMN is created by the primary tumor and can acquire permissive and supportive properties allowing for tumor colonization at target organs [24, 25]. The role of lipid reprogramming in PMN formation remains elusive. In this study, we found that ACACA inhibition played a major role in the formation of the lung PMN in breast cancer, and that pathologically reduced levels of ACACA in lung fibroblasts induced lipid metabolism abnormalities, fibroblast senescence and inflammation, resulting in breast cancer metastasis.

The altered lipid metabolism in cancer cells or their surrounding niche involves a series of altered enzyme activities. This has been associated with the metastatic capacity of various cancers [26, 27], however its role in the formation of the PMN is poorly understood. Recently, Zhang et al. reported that colon cancer cells changed lipid metabolism of fibroblasts by phosphorylating ATP-citrate lyase, which upregulated the level of acetyl-CoA in hepatic PMN [28]. Oxysterols, endogenous regulators of lipid metabolism, were shown to modify the lung metastatic niche in an orthotopically implanted 4T1 breast tumor mouse model [29]. Bone marrow adipocytes have been shown to prevent the spontaneous apoptosis of acute monocytic leukemia cells by regulating their metabolic energy balance, and the disruption of fatty acid β-oxidation in bone marrow adipocytes has been shown to increase cancer cell death [30]. Reprogramming of lipid profile has also been found in cancer-associated fibroblasts and macrophages, and this has been shown to potentiate cancer progression [31, 32]. In this study, we observed a different pattern of lipidomics in the lungs of MMTV-PyVT mice. The change in lipid profile was caused by the downregulation of several lipid metabolism enzymes in the lungs of breast cancer-bearing mice. Several phospholipids, including PC, PE and SM were decreased in lung tissues, implying changes in the membranes of the cells in the lungs of the tumor-bearing mice. However, little is known about lipid alterations in the components of the PMN, and how this metabolic reprogramming affects lung metastasis of breast cancer cells. Further studies are needed to investigate this issue.

The role of ACACA in oncology is controversial. It has been considered to act as an oncogene, since inhibition of ACACA by siRNA or chemical inhibitors has been shown to decrease cell growth in prostate and non-small cell lung cancer. In contrast, blockade of ACACA has been shown to induce epithelial-mesenchymal transition and metastasis by increasing cellular acetyl-CoA and Smad2 acetylation [33]. Cancer-related metabolic reprograming is not limited to tumor cells, but is also affected by non-malignant cells. Inhibition of ACACA has been shown to induce cell cycle arrest and senescence in proliferative fibroblasts [20]. Senescent fibroblasts are considered to promote cancer development by remodeling a permissive metabolic microenvironment via secretion of metabolites such as lactate, ketones, glutamine, nitric oxide and reactive oxygen species into the microenvironment [34]. Recently, Nicolas et al. reported that inflammatory cancer-associated fibroblasts with the p53-mediated therapy-induced senescence phenotype contributed to a poor response to chemoradiotherapy [35]. To the best of our knowledge, the present study is the first to demonstrate a decreased expression of ACACA in fibroblasts of lung PMN, which subsequently caused acetyl-CoA accumulation and excess acetylated modification in total proteins, resulting in cell senescence. Furthermore, induction of ACACA expression in the lungs prevented lung metastasis of breast cancer in vivo. This suggests that ACACA may be a critical factor associated with lung PMN formation and may be a therapeutic target for breast cancer.

The regulation and improvement of the tumor immune microenvironment are widely recognized to play an important role in cancer therapy [36, 37]. The PMN has been shown to consist of various mature and immature immune cells, such as bone marrow-derived hematopoietic progenitor cells, MDSCs, macrophages and neutrophils, which accumulate in the pre-metastatic microenvironment of organs different to the primary site before the arrival of cancer cells [38–40]. In mice, MDSCs are characterized by the surface expressions of CD11b and Gr-1, and are further divided into monocytic (CD11b^+^Ly6C^high^Ly6G^−^) (M-MDSCs) and polymorphonuclear (CD11b^+^Ly6C^low^Ly6G^+^) MDSCs (G-MDSCs or PMN-MDSCs) [39]. MDSCs can increase the survival and metastatic potential of circulating tumor cells (CTCs) by soluble factors or cell–cell contact. MDSCs can protect CTCs in the circulation from a hostile microenvironment and assist CTC extravasation by secreting reactive oxygen species [41]. Furthermore, PMN-MDSCs (as known as G-MDSCs) can directly interact with CTCs to form a CTC/PMN-MDSC complex, and enhance their pro-tumorigenic ability [42]. The mechanisms underlying the infiltration of MDSCs by lung host cells in the PMN have not been fully established. Lung epithelial cell-derived C–C motif chemokine ligand 2 (CCL2) has been reported to recruit MDSCs and contribute to the lung PMN establishment [43]. Lung endothelial cells and alveolar macrophages were also shown to contribute to Mac1^+^-myeloid cell or BMDC recruitment to the lungs by releasing S100A8 in melanoma mouse model [44]. In our study, we found that senescent pulmonary fibroblasts contributed to G-MDSCs recruitment by generating an inflammatory microenvironment via CXCL1 upregulation. CXCL1 increased the movement of G-MDSCs, whereas neutralizing CXCL1 with antibodies prevented the senescent lung fibroblast-mediated movement of G-MDSCs. Taken together, these findings suggest that CXCL1 may be an important target for inhibiting the pre-metastatic lung microenvironment to suppress lung metastasis in breast cancer.

## Conclusions

In conclusion, our results indicate that breast cancer alters lipid metabolism of fibroblasts to establish a PMN to promote lung metastasis. Mechanistically, ACACA downregulation converts fibroblasts to the senescence and inflammation phenotype in the lung PMN. Meanwhile, increased secretion of CXCL1 from lung fibroblasts reduces the immunity of the lung microenvironment through the recruitment of G-MDSCs (Fig. 7E). Our study not only demonstrates a novel molecular mechanism of PMN formation, but also provides clinical insights into the therapeutic implication of ACACA and CXCL1 to prevent lung metastasis in breast cancer.

## Supplementary Information

Below is the link to the electronic supplementary material.

Fig. S1 The H&E staining of the lungs of MMTV-PyVT mice at different weeks of age (12th to 15th week).(PNG 1.90 mb)

High Resolution (TIF 5.50 mb)

Fig. S2 The pathway analysis of gene profile of lungs of MMTV-PyVT mice. (A) GO_BP pathway. (B) GO_MF pathway. The expression of lipid metabolism-related genes in (C) lung epithelial cells and (D) lung fibroblasts after co-cultured with mouse primary breast cancer (PBC). Lung fibroblasts were isolated from the lungs of wild-type mice, and mouse PBCs were isolated from primary mammary glands of MMTV-PyVT mice. The lung epithelial cell line MEL-12 or lung fibroblasts were co-cultured with PBC in a transwell system for 48 h, and the expression of various genes in lung epithelial cells or fibroblasts was assessed by qRT-PCR. Graphs show mean ± SD. *, P < 0.05; **, P < 0.01.(PNG 841 kb)

High Resolution (TIF 3.36 mb)

Fig. S3 Typical total ion chromatography (TIC) of lipid profiles. (A) TIC of lungs of wild-type (WT) mice. (B) TIC of lungs of MMTV-PyVT mice. (PNG 523 kb)

High Resolution (TIF 2.03 mb)

Fig. S4 The senescence phenotype of lung of MMTV-PyVT mice. (A-C) The senescent phenotype of lung of mice at 9th to 11th week. The expression of Ki67 (D) pR6S (E), and p27 (F) in the fibroblast co-cultured with MCF7 and Hs 578T. The Ki67 (G) pR6S and p27 (H) in the human lung fibroblast transfected with ACACA siRNA. (I) The level of CXCL1 in the lung fibroblasts isolated from mice at 9th week. Graphs show mean ± SD. *, P < 0.05; **, P < 0.01.(PNG 576 kb)

High Resolution (TIF 2.44 mb)

Fig. S5 Senescent fibroblasts increased cancer progression. The condition medium (CM) of breast cancer-derived fibroblasts increased (A) cell proliferation and (B) migratory ability of MCF7, Hs 578T and PBC cells. Lung fibroblasts isolated from the lungs of wild-type mice were co-cultured with PBC for 5 days and the supernatants were collected as condition medium (CM). The human lung fibroblasts were co-cultured with MCF7 or Hs 578T for 5 days. After washing, fresh medium was added and the media were harvested to act as CMs after 24 h incubation. The effect of these CMs on cell proliferation was assessed using WST-1 analysis (for 48 h incubation), while migration of cells was assessed in a transwell system for 48 h. Graphs show mean ± SD. *, P < 0.05; **, P < 0.01.(PNG 183 kb)

High Resolution (TIF 1.13 mb)

Fig. S6 The infiltration of m-MDSCs and Treg. The cell number of (A) M-MDSCs and (B) Treg in the lungs of mice at 9th week. ns, not significant.(PNG 195 kb)

High Resolution (TIF 1.20 mb)

Table S1 (PDF 86 kb)

Table S2 (PDF 93 kb)

Table S3 (PDF 116 kb)

## Data Availability

The data that support the findings of this study are available from the corresponding author upon reasonable request.

## Abbreviations

MG: 2-Monoglyceride
ACACA: Acetyl-CoA carboxylase alpha
BMDCs: Bone marrow-derived cells
CAFs: Cancer-associated fibroblasts
PMN: Pre-metastatic niche
CE: Cholesteryl ester
CR: Ceramide
CL: Cardiolipin
DG: Diacylglycerol
CM: Condition medium
Hex2Cer: Dihexosylceramide
HexCer: Hexosylceramide
IHC: Immunohistochemistry
PBC: Primary breast cancer
PA: Phosphatidic acid
PC: Phosphatidylcholine
PE: Phosphoethanolamine
PG: Phosphatidylglycerol
PI: Phosphatidylinositol
PS: Phosphatidylserine
SM: Sphingomyelin
SP: Sphingosine
TME: Tumor microenvironment
TG: Triglyceride
TIC: Typical total ion chromatography
WT: Wild-type
