# Supplementary figures and images for: Involvement of ACACA (acetyl-CoA carboxylase α) in the lung pre-metastatic niche formation in breast cancer by senescence phenotypic conversion in fibroblasts
Source: Cell Oncol (Dordr). 2023 Jan 6;46(3):643–60. doi: 10.1007/s13402-022-00767-5 (PMC10205862; doi:10.1007/s13402-022-00767-5)

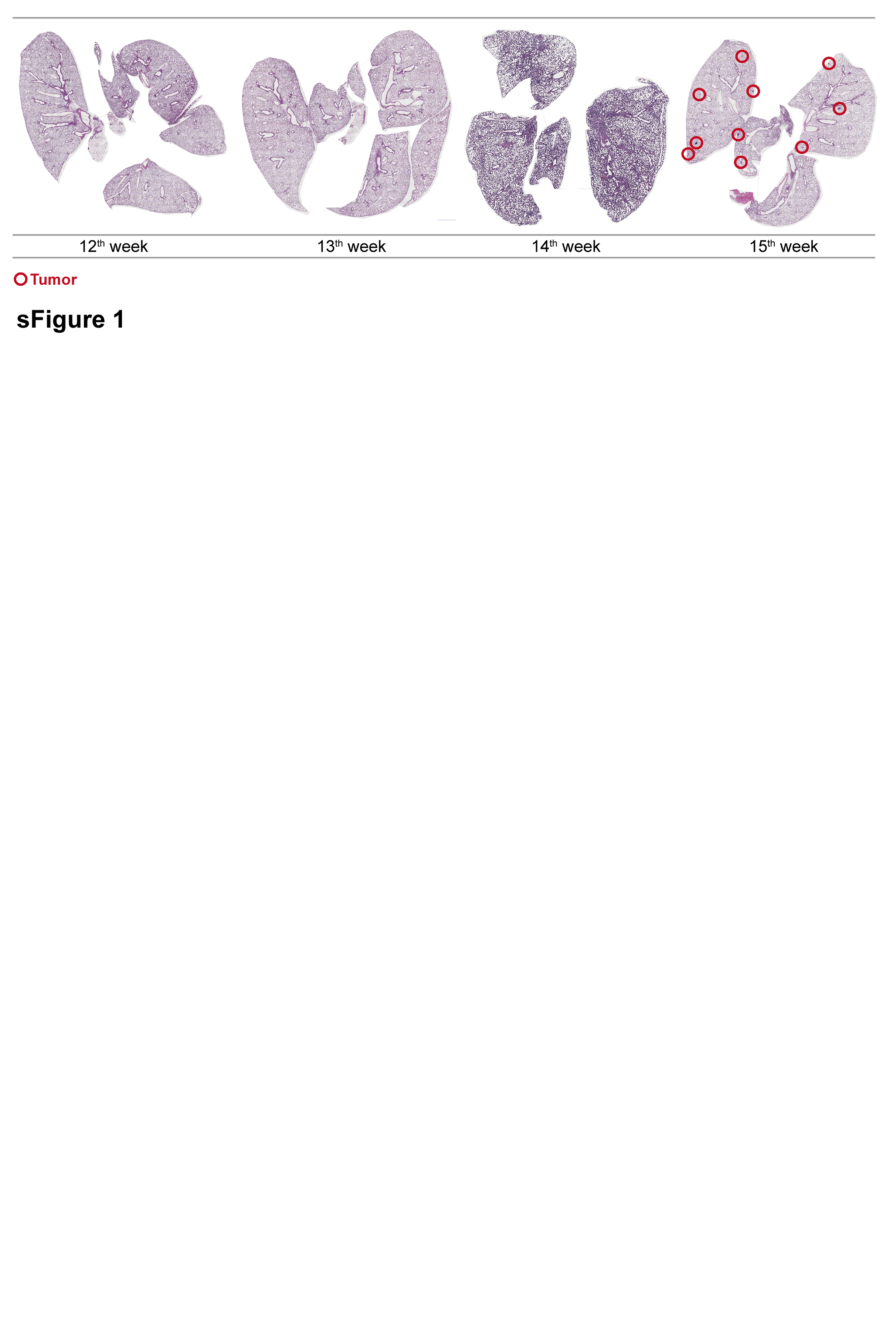

Supplement: Supplementary file 1 — The H&E staining of the lungs of MMTV-PyVT mice at different weeks of age (12th to 15th week).(PNG 1.90 mb) [file 13402_2022_767_Fig8_ESM.png]

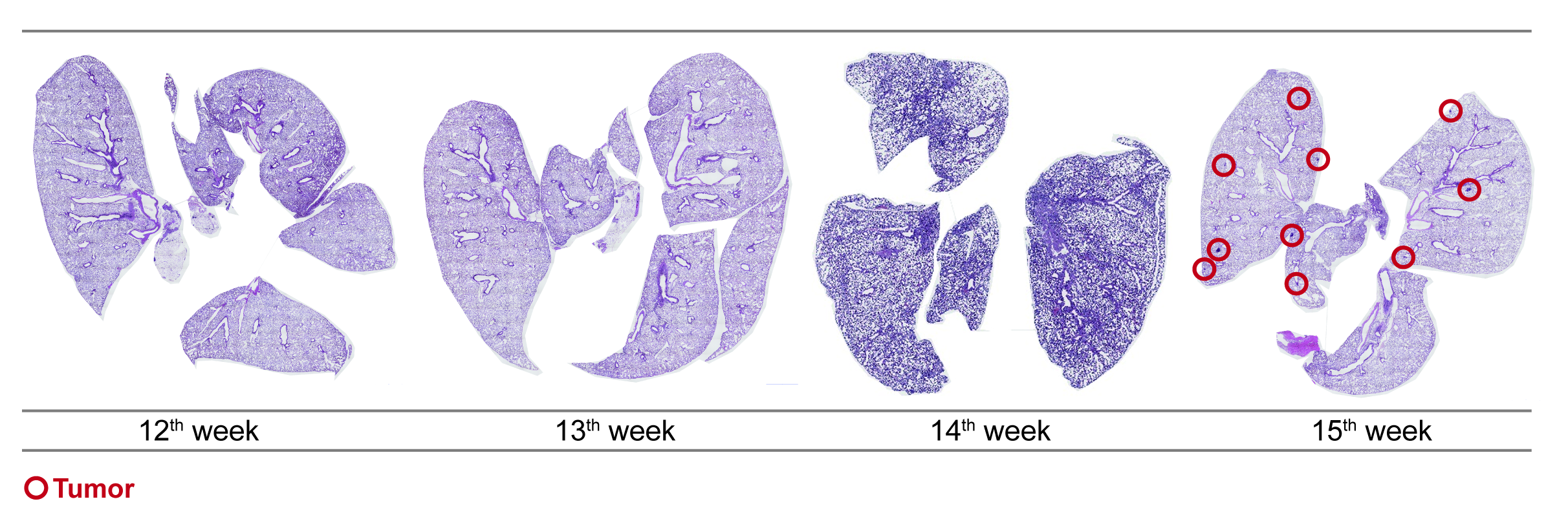

Supplement: Supplementary file 2 — High Resolution (TIF 5.50 mb) [file 13402_2022_767_MOESM1_ESM.tif]

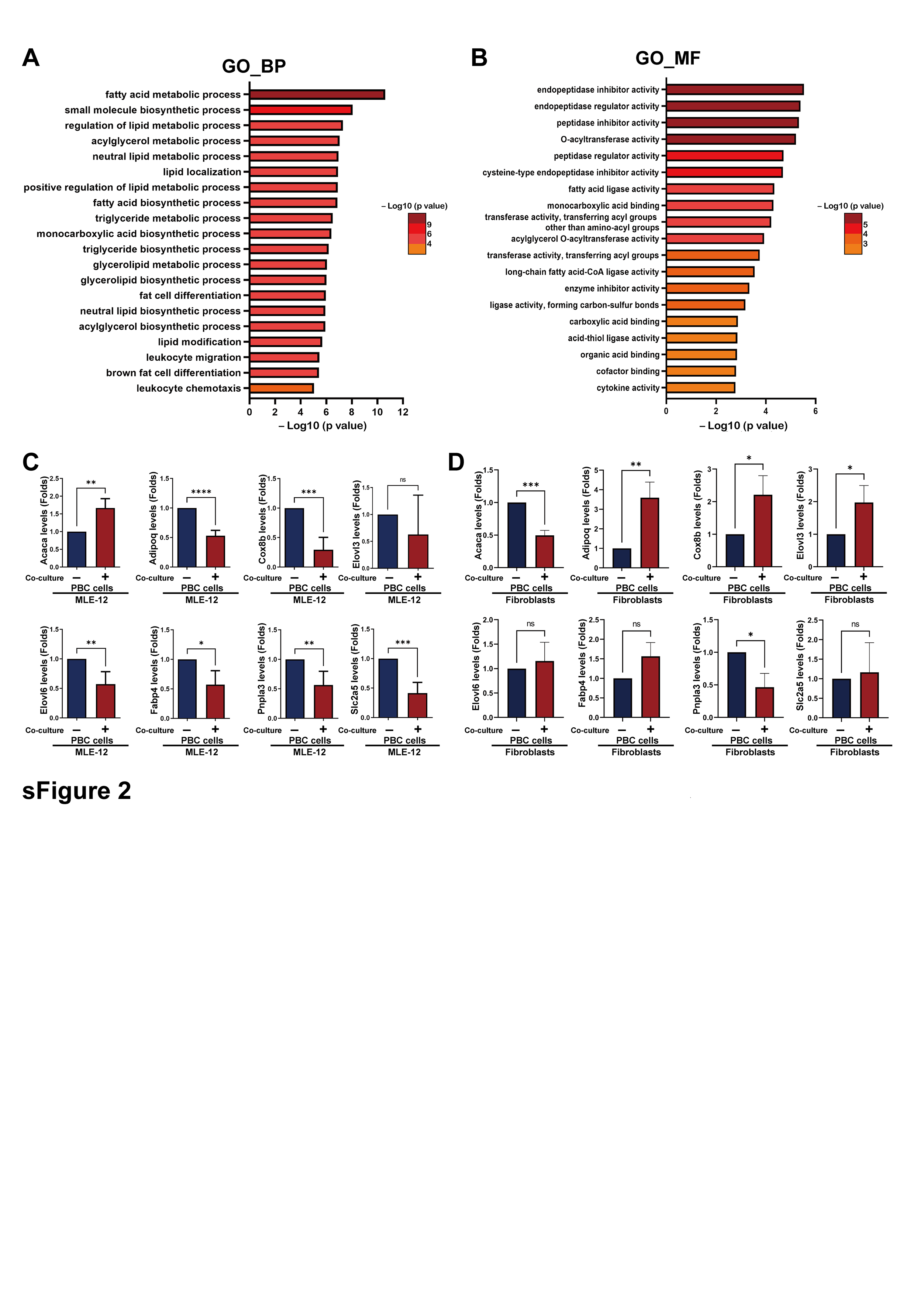

Supplement: Supplementary file 3 — The pathway analysis of gene profile of lungs of MMTV-PyVT mice. (A) GO_BP pathway. (B) GO_MF pathway. The expression of lipid metabolism-related genes in (C) lung epithelial cells and (D) lung fibroblasts after co-cultured with mouse primary breast cancer (PBC). Lung fibroblasts were isolated from the lungs of wild-type mice, and mouse PBCs were isolated from primary mammary glands of MMTV-PyVT mice. The lung epithelial cell line MEL-12 or lung fibroblasts were co-cultured with PBC in a transwell system for 48 h, and the expression of various genes in lung epithelial cells or fibroblasts was assessed by qRT-PCR. Graphs show mean ± SD. *, P < 0.05; **, P < 0.01.(PNG 841 kb) [file 13402_2022_767_Fig9_ESM.png]

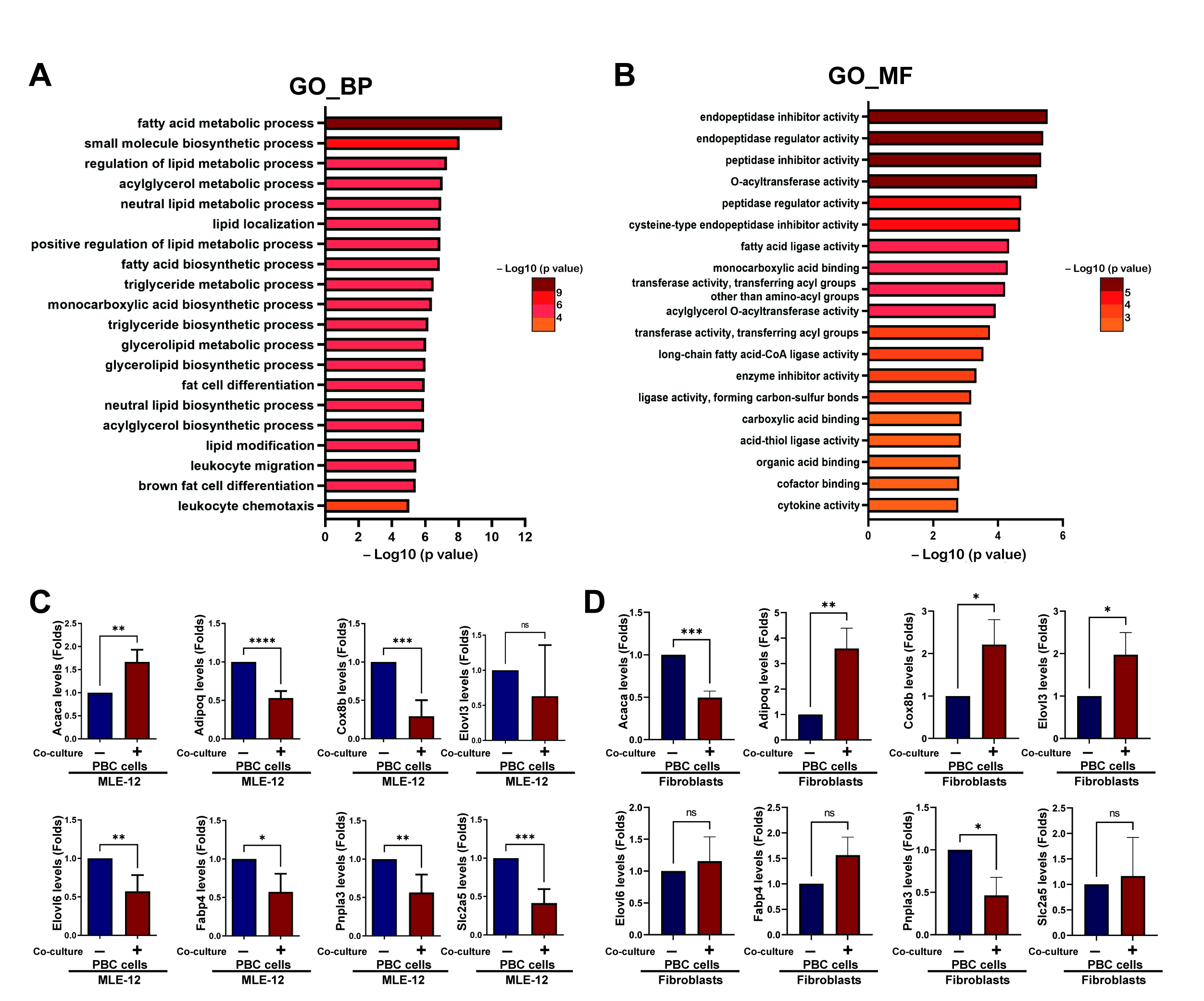

Supplement: Supplementary file 4 — High Resolution (TIF 3.36 mb) [file 13402_2022_767_MOESM2_ESM.tif]

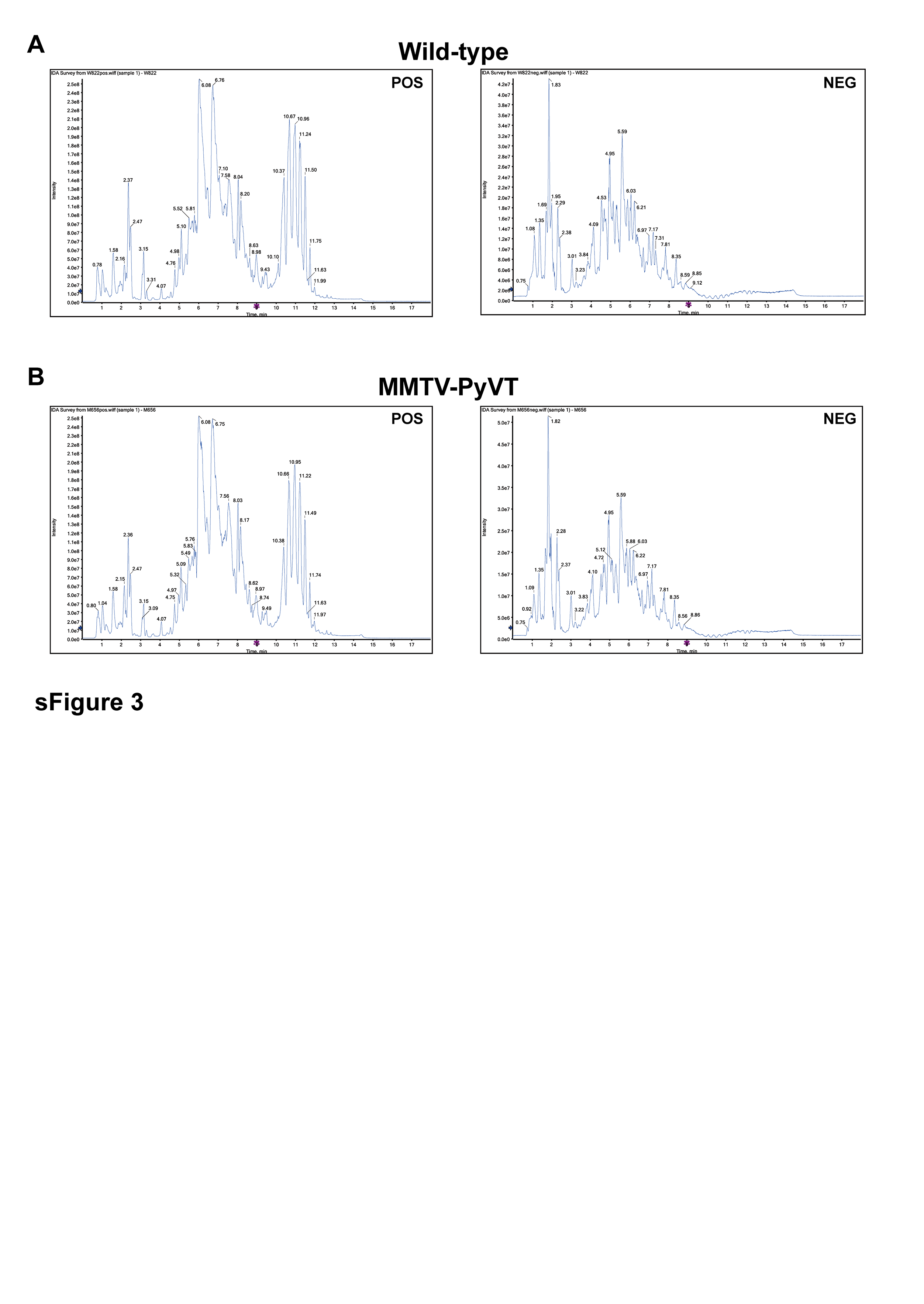

Supplement: Supplementary file 5 — Typical total ion chromatography (TIC) of lipid profiles. (A) TIC of lungs of wild-type (WT) mice. (B) TIC of lungs of MMTV-PyVT mice. (PNG 523 kb) [file 13402_2022_767_Fig10_ESM.png]

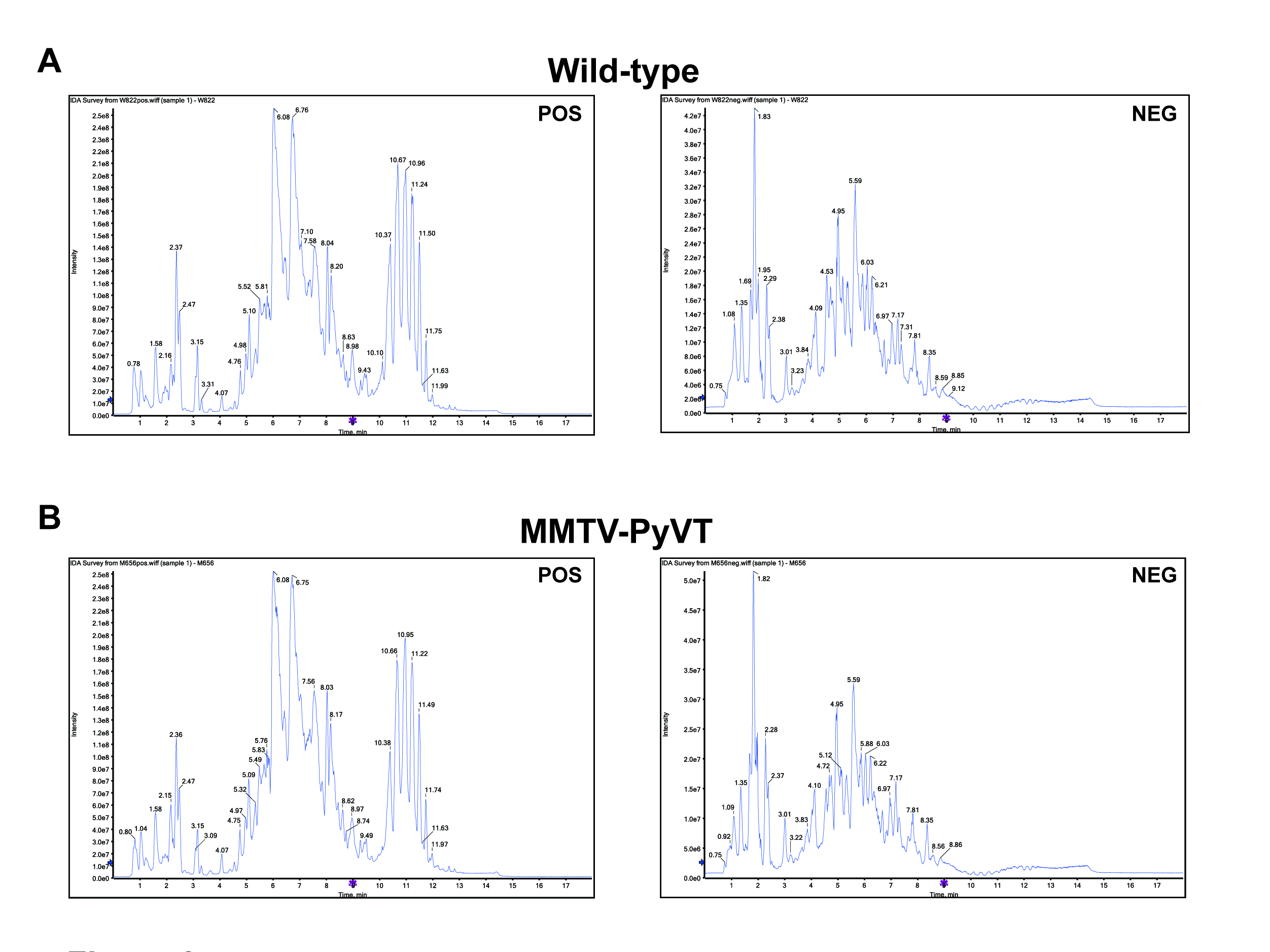

Supplement: Supplementary file 6 — High Resolution (TIF 2.03 mb) [file 13402_2022_767_MOESM3_ESM.tif]

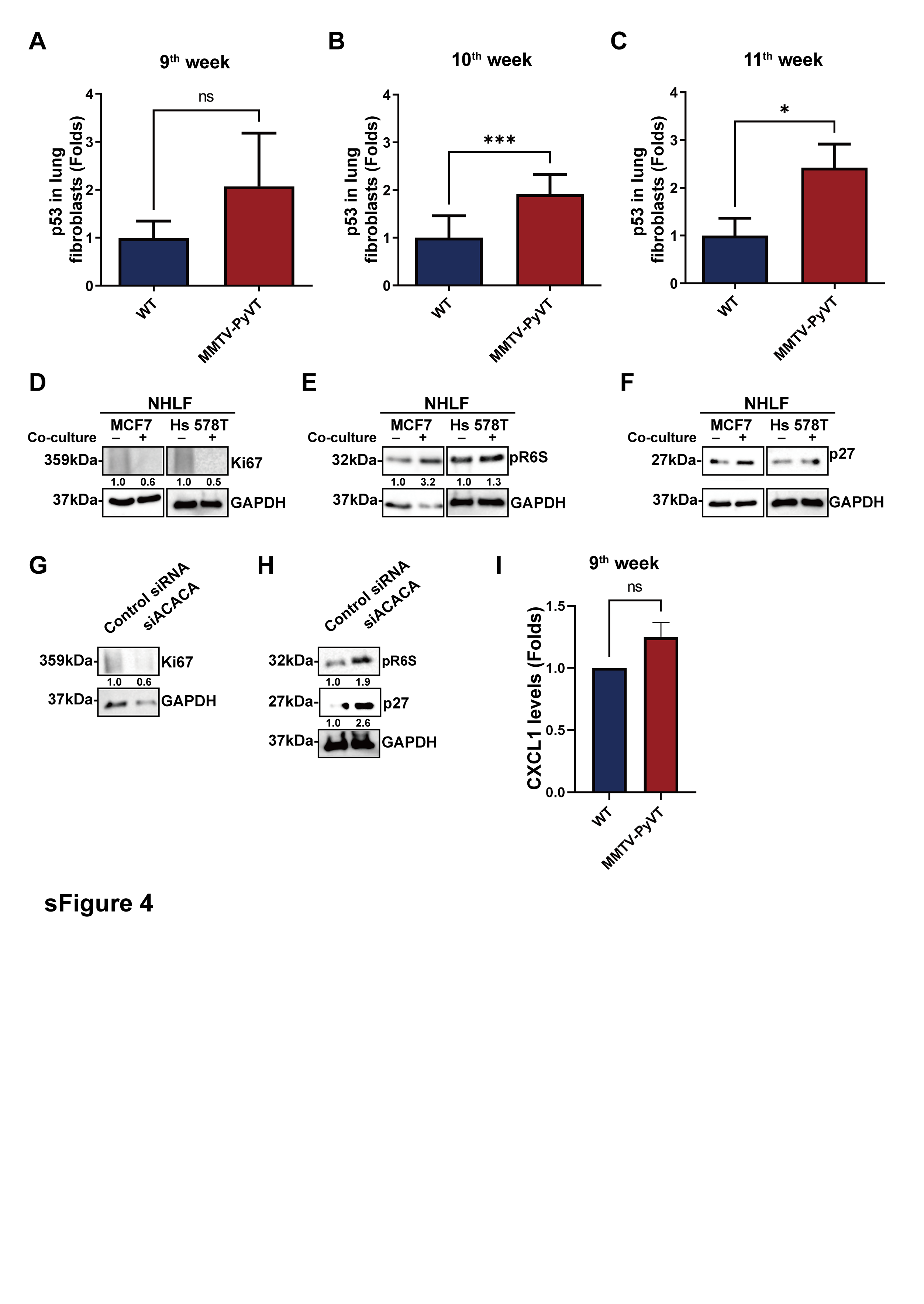

Supplement: Supplementary file 7 — The senescence phenotype of lung of MMTV-PyVT mice. (A-C) The senescent phenotype of lung of mice at 9th to 11th week. The expression of Ki67 (D) pR6S (E), and p27 (F) in the fibroblast co-cultured with MCF7 and Hs 578T. The Ki67 (G) pR6S and p27 (H) in the human lung fibroblast transfected with ACACA siRNA. (I) The level of CXCL1 in the lung fibroblasts isolated from mice at 9th week. Graphs show mean ± SD. *, P < 0.05; **, P < 0.01.(PNG 576 kb) [file 13402_2022_767_Fig11_ESM.png]

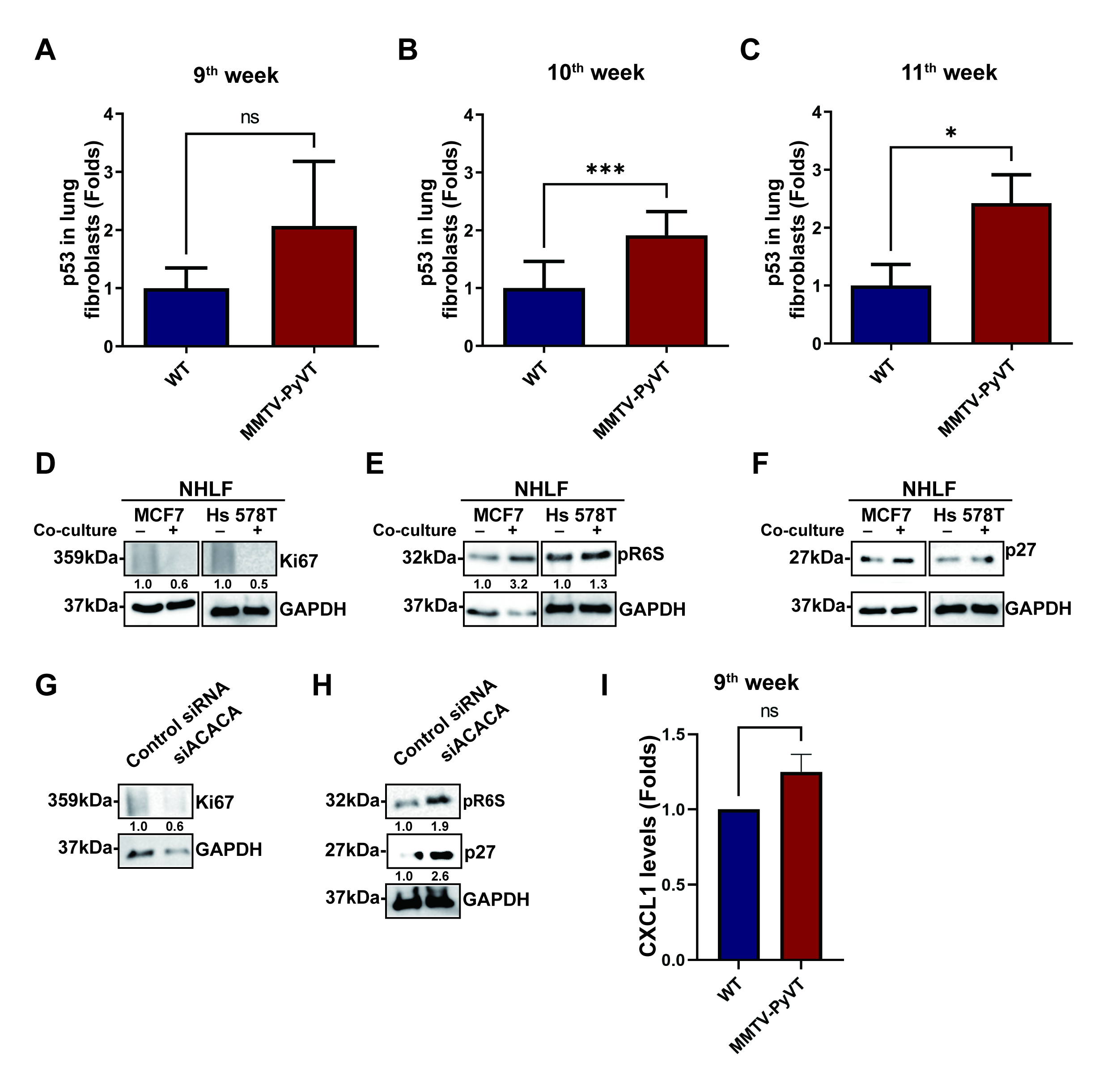

Supplement: Supplementary file 8 — High Resolution (TIF 2.44 mb) [file 13402_2022_767_MOESM4_ESM.tif]

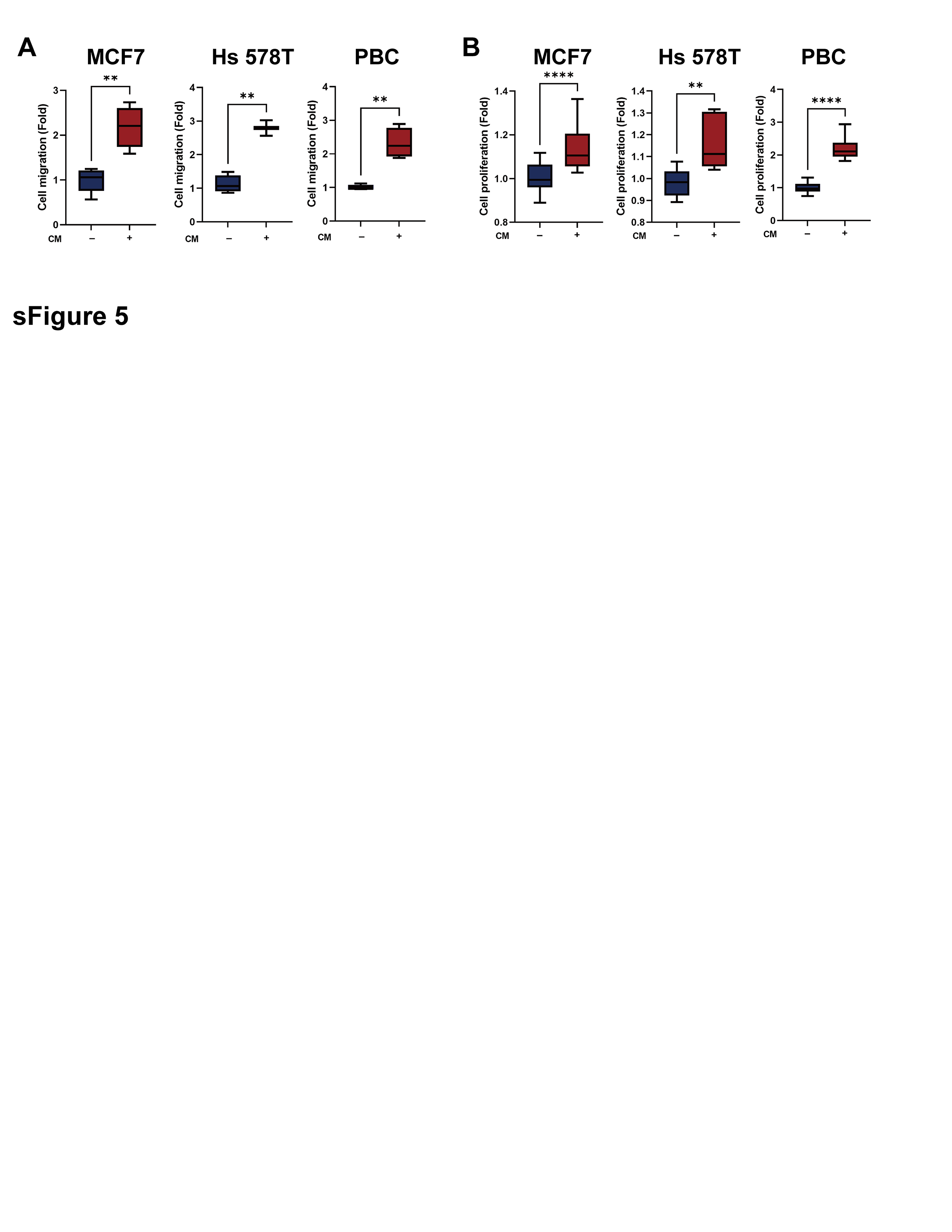

Supplement: Supplementary file 9 — Senescent fibroblasts increased cancer progression. The condition medium (CM) of breast cancer-derived fibroblasts increased (A) cell proliferation and (B) migratory ability of MCF7, Hs 578T and PBC cells. Lung fibroblasts isolated from the lungs of wild-type mice were co-cultured with PBC for 5 days and the supernatants were collected as condition medium (CM). The human lung fibroblasts were co-cultured with MCF7 or Hs 578T for 5 days. After washing, fresh medium was added and the media were harvested to act as CMs after 24 h incubation. The effect of these CMs on cell proliferation was assessed using WST-1 analysis (for 48 h incubation), while migration of cells was assessed in a transwell system for 48 h. Graphs show mean ± SD. *, P < 0.05; **, P < 0.01.(PNG 183 kb) [file 13402_2022_767_Fig12_ESM.png]

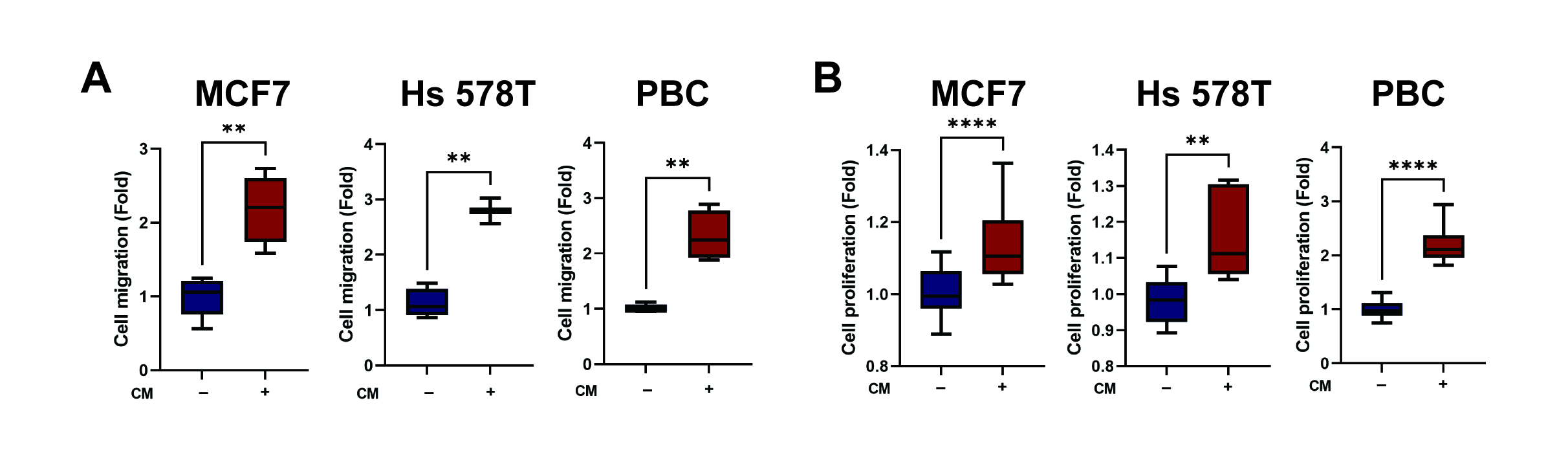

Supplement: Supplementary file 10 — High Resolution (TIF 1.13 mb) [file 13402_2022_767_MOESM5_ESM.tif]

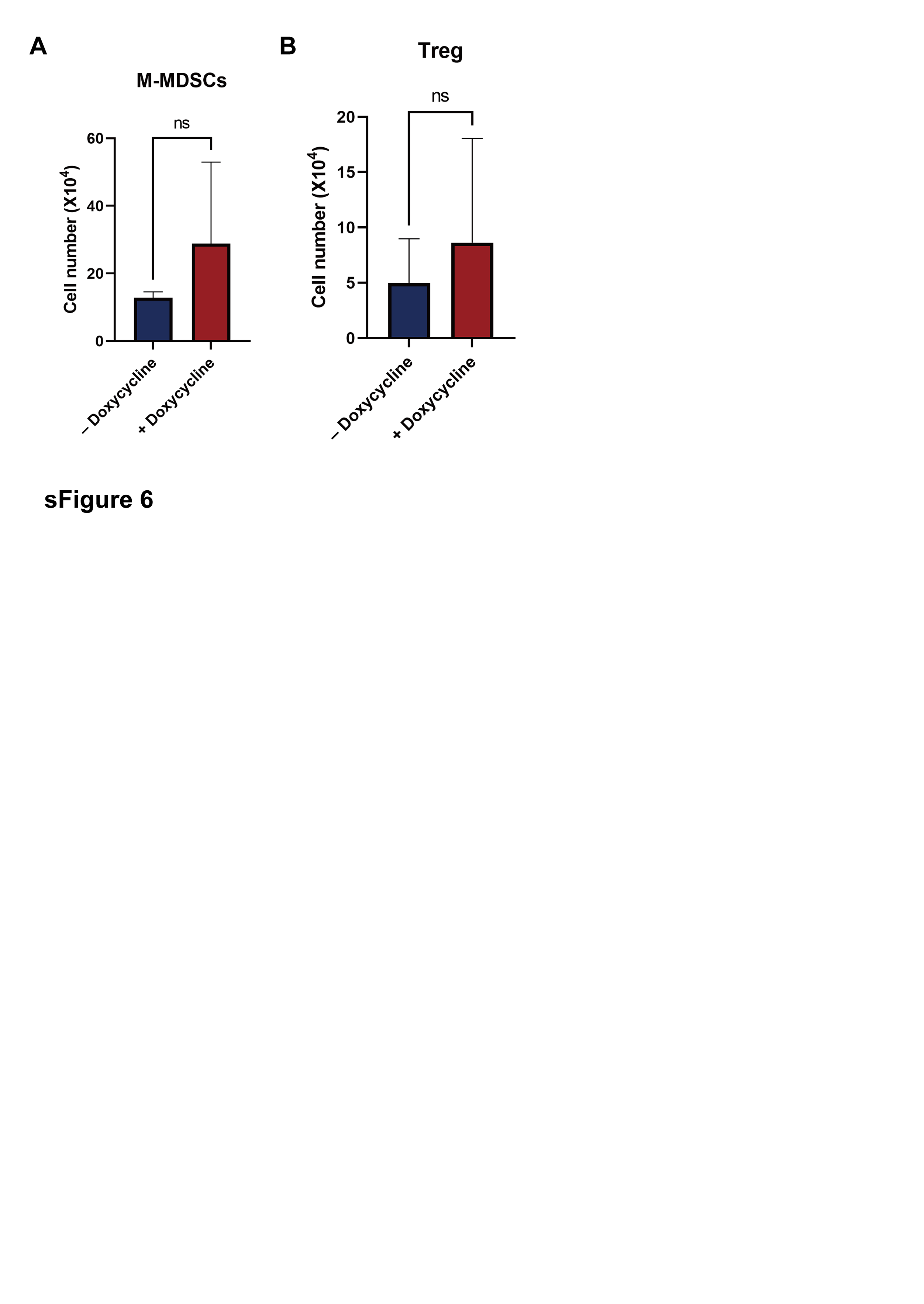

Supplement: Supplementary file 11 — The infiltration of m-MDSCs and Treg. The cell number of (A) M-MDSCs and (B) Treg in the lungs of mice at 9th week. ns, not significant.(PNG 195 kb) [file 13402_2022_767_Fig13_ESM.png]

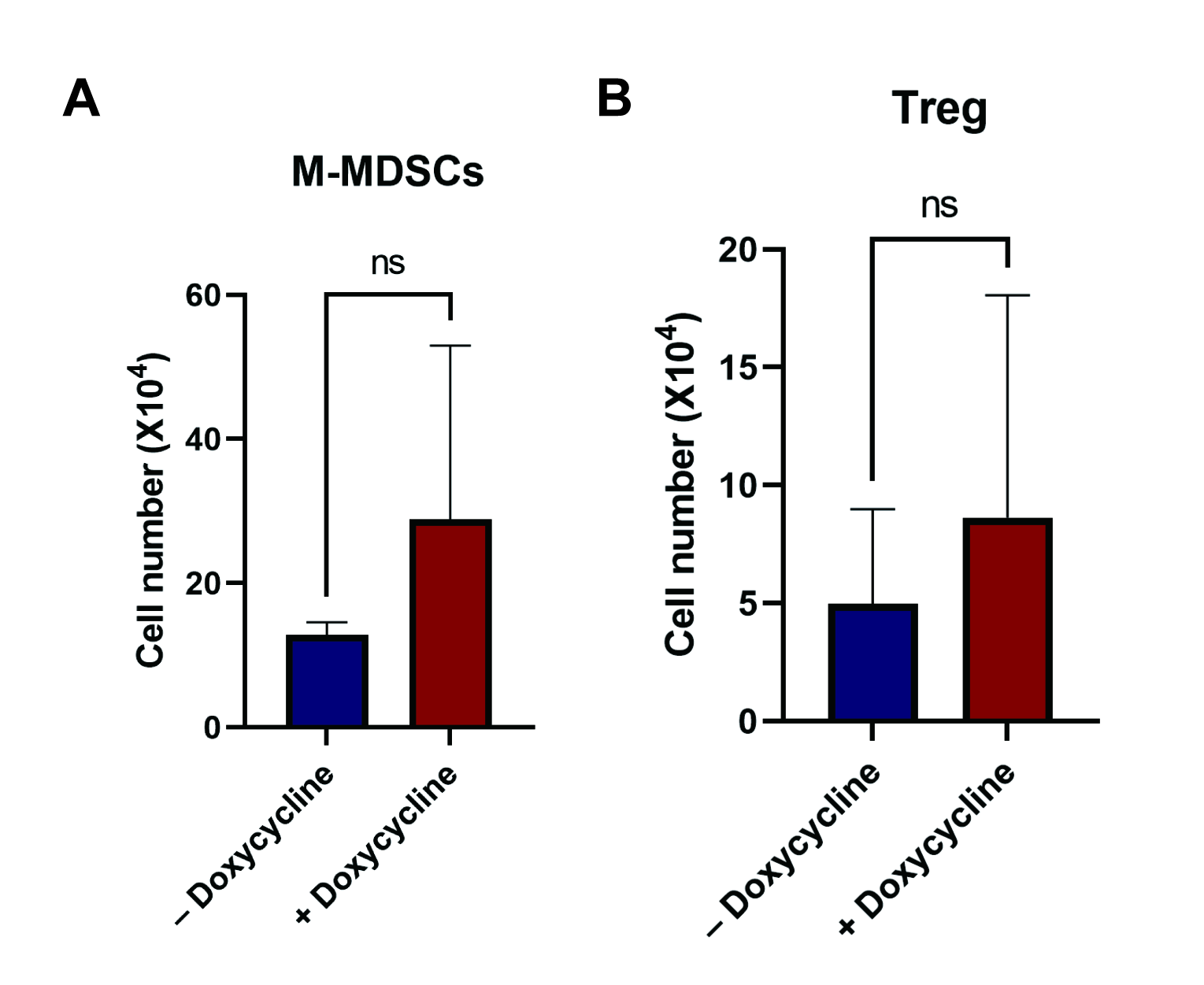

Supplement: Supplementary file 12 — High Resolution (TIF 1.20 mb) [file 13402_2022_767_MOESM6_ESM.tif]
