# Supplementary material for: Involvement of ACACA (acetyl-CoA carboxylase α) in the lung pre-metastatic niche formation in breast cancer by senescence phenotypic conversion in fibroblasts
Source: Cell Oncol (Dordr). 2023 Jan 6;46(3):643–60. doi: 10.1007/s13402-022-00767-5 (PMC10205862; doi:10.1007/s13402-022-00767-5)
Supplement: Supplementary file 13 — (PDF 86 kb) [file 13402_2022_767_MOESM7_ESM.pdf]

sTable 1, The primers used in this study

Gene	Primer
Adipoq	Forward : 5'-TGATGGCAGAGATGGCACTC-3'
	Reverse: 5'-ACACATAAGCGGCTTCTCCA-3'
Acaca	Forward : 5'-ATGGGCTGCTTCTGTGACTC-3'
	Reverse: 5'-CTGCAAGCCTGTCATCCTCA-3'
Elovl6	Forward : 5'-ATGACCAAAGGCCTGAAGCA-3'
	Reverse: 5'-ACAGGAGCACAGTGATGTGG-3'
Elovl3	Forward : 5'-TCGTTGTTGGCCAGACCTAC-3'
	Reverse: 5'-TAGATGGCAAAGCACACGGT-3'
Pnpla3	Forward : 5'-CAGCCTGAGTCTGTCCTTGG-3'
	Reverse: 5'-GGTAGCCCTCCCTGTCCTTA-3'
Cox8b	Forward : 5'-CCTGCGAAGTTCACAGTGGT-3'
	Reverse: 5'-GCTCTCCAAGTGGGCTAAGAC-3'
Fabp4	Forward : 5'-TCACCATCCGGTCAGAGAGT-3'
	Reverse: 5'-CCAGCTTGTCACCATCTCGT-3'
Slc2a5	Forward : 5'-TGCAGCCAAATTGCCCAATC-3'
	Reverse: 5'-GATGAAGAGTTGCGGGACCA-3'
Acsm3	Forward : 5'- ATGGACCCAAGGAGCATGTG-3'
	Reverse: 5'-GGTTCTCCAGCACTGACACA-3'
Slc2a4	Forward : 5'-ACCAACTGGCCATCGTCATT-3'
	Reverse: 5'-AGGTATCTGGGGCTCTCAGG-3'
ACACA	Forward : 5'-GCCTCTTCCTGACAAACGAG-3'
	Reverse: 5'-TTGATTGCAGCAGGTCTTTG-3'
