# Supplementary material for: Involvement of ACACA (acetyl-CoA carboxylase α) in the lung pre-metastatic niche formation in breast cancer by senescence phenotypic conversion in fibroblasts
Source: Cell Oncol (Dordr). 2023 Jan 6;46(3):643–60. doi: 10.1007/s13402-022-00767-5 (PMC10205862; doi:10.1007/s13402-022-00767-5)
Supplement: Supplementary file 14 — (PDF 93 kb) [file 13402_2022_767_MOESM8_ESM.pdf]

**sTable 2. The antibodies used in this study**

| Antibody | Company | Catalogue |
|---|---|---|
| ACACA | Cell Signaling | 4190 |
| α-Tubulin | Cell Signaling | 2125 |
| Actin | Millipore | MAB1510 |
| GAPDH | Millipore | MAB374 |
| Acetylated lysine | Cell Signaling | 9441 |
| p21 (for mouse) | Cell Signaling | 64016 |
| p21 (for human) | Cell Signaling | 2946 |
| P27 | Cell Signaling | 3688 |
| Ki67 | GeneTex | GTX16667 |
| Phospho-S6 Ribosomal Protein (Ser235/236) | Cell Signaling | 2211 |
