# Supplementary material for: Involvement of ACACA (acetyl-CoA carboxylase α) in the lung pre-metastatic niche formation in breast cancer by senescence phenotypic conversion in fibroblasts
Source: Cell Oncol (Dordr). 2023 Jan 6;46(3):643–60. doi: 10.1007/s13402-022-00767-5 (PMC10205862; doi:10.1007/s13402-022-00767-5)
Supplement: Supplementary file 15 — (PDF 116 kb) [file 13402_2022_767_MOESM9_ESM.pdf]

sTable 3. The cytokines in the lung fibroblasts of wild-type and MMTV-PyVT mice

Group (Control)	Factors	Mean±S.D.		Mean±S.D.
Wild-type	GM-CSF	1.00±0.04	MMTV-PyVT	2.86±2.55***
	CCL2	1.00±0.37		13.87±14.41***
	G-CSF	1.00±0.15		51.64±44.30***
Fibroblast	GM-CSF	1.04±0.14	Fibroblast co-culture with PBC cells	1.23±0.16**
	CCL2	0.98±0.31		1.90±1.44***
	G-CSF	1.00±0.24		10.11±8.21***
NHLF	IL-8	1.00±0.00	Fibroblast co-culture with MCF7 cells	2.76±1.63*
NHLF	IL-8	1.00±0.00	Fibroblast co-culture with Hs 578T cells	4.36±3.50
Control siRNA	IL-8	1.00±0.49	siACACA	2.21±0.85***

*, $P < 0.05$; **, $P < 0.01$; ***, $P < 0.001$
